# The impact of the large B-cell lymphoma tumor microenvironment on efficacy of CD19-directed CAR-T immunotherapy

**DOI:** 10.3389/fimmu.2026.1819591

**Published:** 2026-06-24

**Authors:** Helen P. Cashman, Cameron J. Turtle

**Affiliations:** 1 School of Medical Sciences, Faculty of Medicine and Health, University of Sydney, NSW, Australia; 2 Sydney Medical School, Faculty of Medicine and Health, University of Sydney, NSW, Australia; 3 Royal North Shore Hospital, NSW, Australia; 4 Translational Science and Therapeutics Division, Fred Hutchinson Cancer Center, Seattle, WA, United States

**Keywords:** CAR-T therapy, CD19, immune microenvironment, immunotherapy, large B-cell lymphoma, treatment resistance, tumor microenvironment

## Abstract

Large B-cell lymphoma is an aggressive lymphoma subtype with poor outcomes in the relapsed and refractory setting. Despite considerable improvements in long-term survival with CD19 CAR-T immunotherapy, over half of patients do not achieve durable remissions. A deeper understanding of the mechanisms within the tumor microenvironment that drive treatment resistance is critical to informing the development of improved CAR-T therapies that can overcome immunosuppressive elements. In this review, we synthesize current knowledge on how histological, cellular, molecular and spatial features of the large B-cell lymphoma microenvironment influence clinical responses following CD19 CAR-T therapy and highlight key knowledge gaps in the field. We discuss data from clinical trials of emerging novel CAR-T engineering strategies aimed at reprogramming the immunosuppressive tumor microenvironment and underscore the growing need for advanced analytical approaches capable of integrating multimodal, high-dimensional datasets to comprehensively characterize this complex tumor ecosystem.

## 1 Introduction

The term large B-cell lymphoma (LBCL) encompasses a group of B-cell lymphoma histologies within the mature B-cell neoplasms that have an aggressive clinical phenotype, are often treated with similar core therapeutic regimens, and have poor outcomes in the refractory and multiply relapsed settings (1). In the 5^th^ Edition of the World Health Organization Classification of Haematolymphoid Tumors (WHO-HAEM5) (2), there are 18 entities recognized as LBCL, including diffuse large B-cell lymphoma, not otherwise specified (DLBCL, NOS), which incorporates the germinal center B-cell (GCB) and activated B-cell (ABC) cell-of-origin (COO) molecular subtypes (2); primary mediastinal large B-cell lymphoma (PMBCL); T-cell/histiocyte-rich large B-cell lymphoma (THRLBCL); DLBCL/high-grade B-cell lymphoma (HGBL) with MYC and BCL2 rearrangements; and HGBL, NOS, among others (2). A new entity in the updated WHO-HAEM5 classification, transformations of indolent B-cell lymphomas (tiNHL), has emerged as a category distinct from LBCL.

Relapsed or refractory (R/R) LBCL typically portends a poor prognosis (3). Refractory or progressive disease after first line anthracycline-based chemoimmunotherapy (CIT) for DLBCL occurs in approximately 30% of patients and was previously treated with salvage chemotherapy followed by autologous stem cell transplant (ASCT) in eligible patients who demonstrated a good response to salvage therapy. Survival rates were modest (3, 4). Approximately 50% of patients were not suitable candidates for ASCT, prompting an interest in the development of more effective treatment options for these patients (4). CD19-directed chimeric antigen receptor (CAR)-modified T-cell (CAR-T) therapy has dramatically altered the treatment landscape of R/R LBCL and other hematological malignancies (see below, 3.2 Clinical Landscape of CAR-T Therapy for LBCL) (5–13) (14, 15).

CAR-T therapy in the autologous setting involves collection of T-cells from a patient by leukapheresis, followed by *in vitro* genetic modification of the collected T-cells to enable expression of a tumor-targeted CAR. After lymphodepletion (LD) chemotherapy and CAR-T reinfusion, binding of the target antigen by the CAR enables major histocompatibility complex (MHC)-independent CAR-T activation and effector function (16), resulting in lysis of target-bearing tumor cells. CAR-T activation can also lead to immune-mediated adverse events, such as cytokine release syndrome (CRS) and immune effector-cell associated neurotoxicity syndrome (ICANS), as well as immune effector-cell associated hematotoxicity (ICAHT) and increased risk of infection. The CD19 CAR-T products currently approved by the US Food and Drug Administration (FDA) and European Medicines Agency for treatment of R/R LBCL include axicabtagene ciloleucel (axi-cel), lisocabtagene maraleucel (liso-cel) and tisagenlecleucel (tisa-cel) (17–19). These three products incorporate second generation CAR structures comprising an extracellular CD19-binding single chain variable fragment (scFv), a hinge domain, a transmembrane domain, and intracellular 4-1BB or CD28 costimulatory sequences with a CD3ζ signaling domain. CAR construct designs and CAR-T manufacturing processes differ between axi-cel, tisa-cel and liso-cel (20), which in part accounts for observed differences in efficacy and toxicities between these products.

Despite improvements in outcomes of R/R LBCL since the introduction of CD19 CAR-T therapy, over half of patients do not achieve durable remission. Numerous factors have been associated with failure, including inherent patient and disease characteristics, CAR-T product attributes, and features of the tumor microenvironment (TME) (20–23). New high-dimensional research techniques have begun to enable a deeper understanding of the lymphoma TME and revealed immuno-biological features of the tumor that may affect disease trajectory and CIT treatment response (24). To improve outcomes of CAR-T therapies, it is imperative that our understanding of the immune milieu of the TME continues to grow. In contrast to prior reviews that have focused predominantly on the LBCL TME outside of CAR-T therapy or technological advances to optimize CAR-T therapies, this review comprehensively examines how histological, cellular, molecular, and spatial determinants of the TME shape clinical responses to CAR-T therapy in LBCL (24–26).

## 2 The immune composition of the LBCL TME

**Figure 1:**
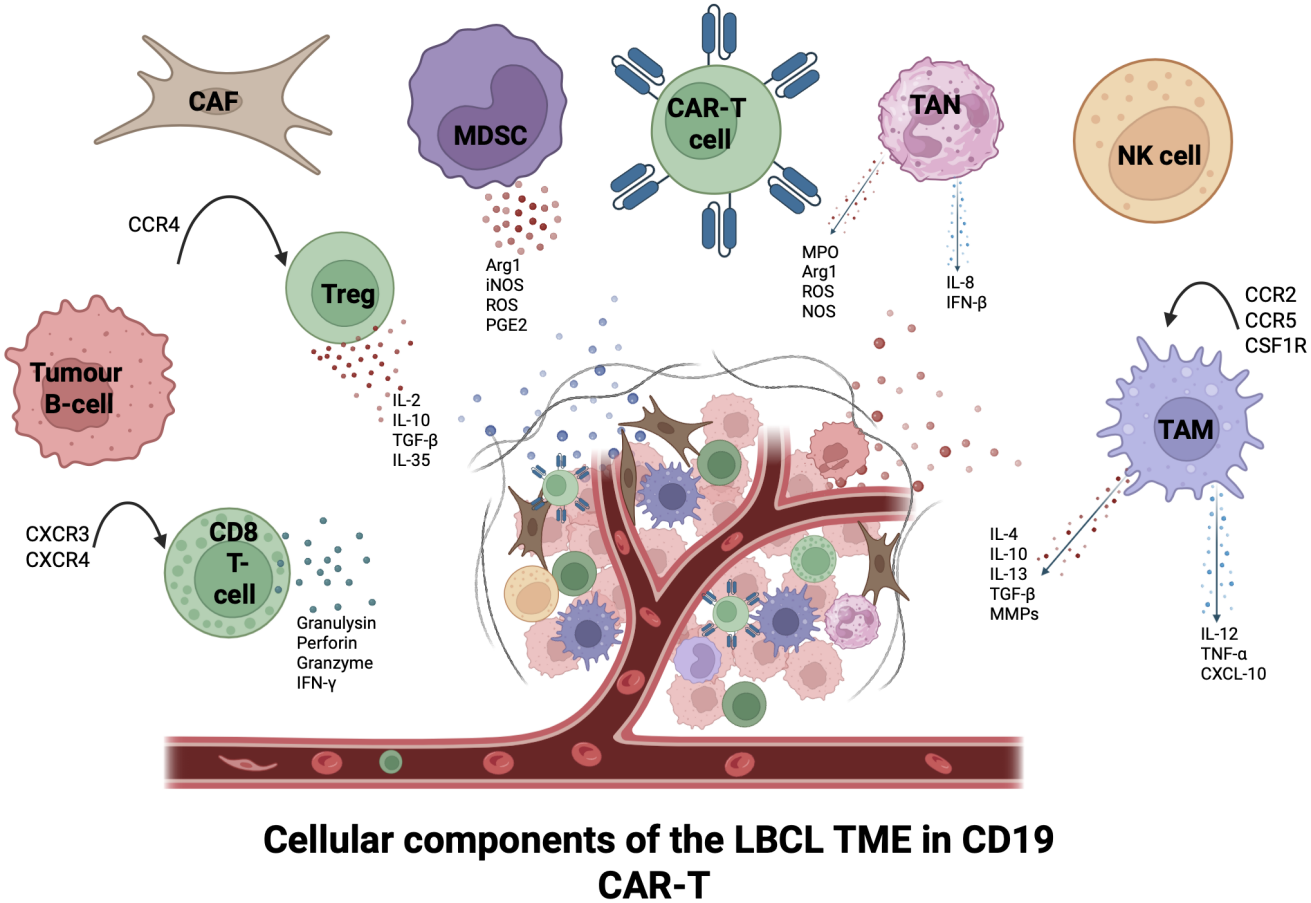
Cellular components of the LBCL TME in CD19 CAR-T therapy. Overview of cell types and molecular interactions within the LBCL tumor microenvironment. T cells, MDSCs, TANs and TAMs are shown with representative pro-inflammatory and anti-inflammatory mediators released into the TME. Rounded arrows indicate recruitment pathways mediated by cell-surface receptors. Treg, regulatory T cell; CAF, cancer-associated fibroblast; MDSC, myeloid-derived suppressor cell; TAN, tumor-associated neutrophil; NK cell, natural killer cell; TAM, tumor-associated macrophage.

The LBCL TME structure is a complex network of cellular and non-cellular components. Cellular components include tumor cells, immune cells (T-cells, tumor associated macrophages (TAMs), dendritic cells (DCs), myeloid-derived suppressor cells (MDSCs), natural killer cells (NK cells), tumor associated neutrophils (TANs)), and stromal cells, including mesenchymal stem cells, cancer associated fibroblasts (CAFs), adipocytes, pericytes and endothelial cells (**Figure 1**) (27). Non-cellular TME components include the extracellular matrix (ECM) and vascular structural elements (26). The immune cell composition and their communications within the TME have important functions in regulating tumor development, surveillance, growth, and metastasis, and can influence response to therapies (28).

Immune cells within the TME can exert tumor promoting or tumor suppressing activities - or both - based on their functions, their effects on the surrounding tumor milieu, and the context. Cytotoxic CD8+ T-cells play a key role in anti-tumor function through the release of the cytotoxic factors granzyme, perforin and granulysin following MHC-I mediated recognition of tumor-associated antigens on cells (28). Activated cytotoxic CD8+ T-cells and CD4+ T helper cells release IFN-γ which induces expression of interferon-stimulated genes involved in cell cycle regulation, inflammatory signaling, apoptosis and transcriptional activation (28, 29). T-cell activation, proliferation, survival and function are regulated by cytokines, such as IL-2, IL-7, IL-12, IL-15, IL-21, GM-CSF and IFNα; chemokine receptors including CXCR3 and CXCR4 recruit CD8+ T-cells to the tumor, whereas CCR4 mediates CD4+ regulatory T-cell (Treg) migration (28, 30). CD4+ Tregs can dampen anti-tumor immune responses through suppression of CD8+ T cells within the TME (26, 31). T-cells can express membrane checkpoint molecules such as PD-1, TIGIT, LAG-3, TIM-3, CTLA-4 and BTLA that bind to corresponding ligands on lymphoma cells, antigen presenting cells, or other cellular components of the TME. The increased expression of these ligands in the LBCL TME attenuates the endogenous anti-tumor immune response through inhibition of T-cell proliferation and effector functions, thereby limiting tumor immunity (32).

Macrophages are another fundamental immune cellular component of the LBCL TME. They have been subclassified, albeit imperfectly, into M1 and M2 subtypes, having anti-tumor and pro-tumor properties, respectively. M2 macrophages have been distinguished from M1 macrophages in the TME by their surface expression of CD163 and CD206; however, macrophages exhibit considerable plasticity and there is no consensus on a definitive distinction of anti-tumor from pro-tumor macrophages in LBCL (33). Macrophages, DCs and other cell types produce Type 1 interferons, which can modulate the function of Tregs, MDSCs, DCs, neutrophils and NK cells to culminate in an anti-tumor response (34). CCR2, CCR5 and CSF1R orchestrate recruitment of TAMs to the TME which can facilitate immunosuppressive macrophage infiltration (30, 35). Macrophages secrete Th2 cytokines (IL-4, IL-10, IL-13), TGF-β, matrix metalloproteinases, as well as CCL17 and CCL22 to modify the inflammatory cytokine environment (35, 36). Further, TAMs can stimulate angiogenesis and breakdown of the ECM to facilitate tumor growth (26). TAM-derived IDO and Arg1 play key immunosuppressive roles in the TME, with IDO promoting MDSC function and both mediators facilitating Treg expansion (26). TGF-β release from tumor cells also recruits TAMs and Tregs to the TME, further amplifying the immunosuppressive effect (26). MDSCs are immature myeloid cells that express high levels of arginase-1 and nitric oxide synthase-2 and have an immunosuppressive effect in the TME (31). NK cells and N1 TANs comprise a small proportion of the immune cells in the TME and tend to display anti-tumor properties (26, 29, 37). DCs are key antigen-presenting cells with important roles in anti-tumor immunity, yet their contribution within the DLBCL tumor microenvironment is comparatively less well characterized.

Non-cellular aspects of the TME that contribute to tumor development and progression include hypoxia, altered metabolic pathways, vascular changes and properties of the ECM. Tumor cells contribute to an immunosuppressive TME by fostering metabolic changes that augment the balance between pro-tumor and anti-tumor responses. Lymphoma cells may preferentially utilize glycolysis as part of the Warburg effect, resulting in a TME characterized by hypoxia, nutrient deprivation and acidic pH (38). Hypoxia influences the TME by impacting metabolic pathways through hypoxia-inducible factor dysregulation, which promotes changes in cell proliferation and drives neoangiogenesis via vascular endothelial growth factor release (39, 40). Increased ECM stiffness within the TME, driven by local soluble factor release and subsequent ECM remodeling, promotes tumor growth by enhancing tumor cell proliferation, suppressing anti-tumor immune responses, reducing the efficacy of cancer therapeutics, and increasing metastatic potential (41). The levels of impact of these factors on the LBCL TME remain unclear.

The function of the lymphoma TME is dependent upon the complex interplay of cellular and non-cellular components that can contribute to tumor development, progression and anti-tumor response, as well as therapeutic susceptibility. Cellular plasticity between anti-tumor and pro-tumor phenotypes through reprogramming during lymphoma development as well as tumor heterogeneity further complicate understanding of these dynamic processes. A range of high dimensional techniques have been employed to study the lymphoma TME in research settings beyond routine clinical laboratory testing, including gene expression profiling, genetic mutational analyses, spatially-focused protein analyses and, in recent years, spatial transcriptomic approaches. The importance of components of the LBCL TME beyond the malignant B-cells has become apparent, with ongoing research enhancing our understanding of the contributions of distinct cell subsets, gene expression profiles (GEP), genetic aberrations, spatial organization and the tumor-immune context to disease biology, prognosis and treatment response.

## 3 Clinical context and predictors of treatment outcomes in LBCL

### 3.1 Associations of the LBCL TME with efficacy of conventional immunochemotherapy

The composition of the immune infiltrate in the LBCL TME associates with response to conventional CIT treatments. TME subtypes have been defined based on the transcriptional landscape of TME cells in LBCL. Steen et al. developed the EcoTyper framework based on bulk and single cell RNA sequencing (RNAseq) data from 1,577 DLBCL patients to classify cell subtypes and transcriptional states within DLBCL tumors, identifying 9 lymphoma ecotypes that demonstrated overall survival (OS) differences following CIT (42). Favorable ecotypes were enriched with GCB lymphomas and fibroblasts, whereas adverse ecotypes were enriched with ABC DLBCL and double hit lymphomas. In a large study incorporating 4,655 DLBCL patients across multiple independent cohorts utilizing bulk RNAseq, Kotlov et al. described 4 LBCL TME categories, termed germinal-center like, mesenchymal, inflammatory and depleted subtypes (43). The depleted subtype, characterized by proliferating tumor cells with low activity of other microenvironmental cells, was associated with the worst progression-free survival (PFS) and OS following rituximab-containing first-line CIT. Furthermore, TME composition changed during lymphoma evolution, with less T-cell infiltration noted at progression (43). In a study of 217 newly diagnosed or R/R DLBCL patients using single nucleus RNAseq, Li et al. identified 3 lymphoma archetype profiles, termed LymphoMAPs, with distinct cell subset compositions and clinical outcome associations (44). A subtype rich in macrophages and CAFs with a deficiency of T-cells occurred more frequently at later lines of therapy; and patients with this lymphoma archetype had inferior PFS following first-line rituximab-based CIT. In a study by Lenz et al, gene expression signatures (GESs) of non-malignant cells were identified that associated with survival following CHOP-based therapy (45). The stromal-1 signature was found in a microenvironment comprised of monocytic cells and active extracellular matrix deposition and associated with superior survival, while the stromal-2 signature had features of increased angiogenesis and associated with inferior survival.

In addition to transcriptional TME profiles, the activation and functional state of immune cell subtypes along with their interactions within the TME may influence LBCL treatment outcomes. In T-cell inflamed primary DLBCL tumors, a low proportion of macrophages was associated with less aggressive disease with better OS and PFS following CIT (46). In this T-cell inflamed TME subtype, shorter OS and PFS were associated with a high proportion of PD-L1+/TIM3+/CD163- macrophages. High TIM-3 expression on TME T-cells was associated with inferior survival in two clinical trials of high-risk primary DLBCL patients treated with intensified CIT including central nervous system prophylaxis (47). High PD-1 expression on CD8+ T-cells and high PD-L1 expression on CD68+ macrophages and T-cells were associated with poorer OS and PFS in *de novo* DLBCL patients treated with rituximab-based CIT (48). Shorter survival in DLBCL has also been associated with a higher density of pretreatment CD163+ M2 TAMs (49). Pre-treatment SPARC protein expression on macrophages has been associated with better survival in DLBCL (45).

Armed with increasingly high-plex antibody marker panels to identify single cells within histological sections, the focus of many researchers has turned to studying the impact of the spatial organization of the DLBCL tumor on disease biology and clinical outcomes. Following on from the previously described TME subtypes derived from GEPs, the DLBCL TME has also been subcategorized into microenvironment categories based on the spatial composition of the immune milieu (50, 51). Reiss et al. performed multiplexed ion beam imaging (MIBI) using 33 markers on two DLBCL cohorts to identify 8 cell neighborhood clusters and 4 cell neighborhood subtypes distinguished by the tumor and immune cell composition (52). Enrichment with CD68+/CD163+ dual-positive macrophages was associated with shorter PFS. In a recent multiplex immunofluorescence study using 12 markers in 99 primary DLBCL patients, Autio et al. examined the prognostic significance of interactions between recurrent cell neighborhood (RCN) subtypes (53). Longer survival was associated with close proximity of CD8+ T-cell rich RCNs to immune poor RCNs, whereas close proximity of CD8+ T-cell rich RCNs to PD-L1+ B-cell rich RCNs was associated with poorer outcomes. These studies have delineated distinct subtypes of LBCL tumors, each characterized by unique immune cell compositions and spatial arrangements that may impact prognosis.

Despite methodological variability across TME studies, consistent themes have emerged, highlighting the importance of T-cell infiltration and the potential adverse prognostic impact of immunosuppressive or pro-tumor cell populations. Reduced overall immune cell presence or specific macrophage co-localization patterns may correlate with inferior prognosis after conventional CIT. New approaches will be needed to uncover the interactions between lymphoma cells and the immune composition of the LBCL TME before developing new TME-targeted therapies to enhance outcomes.

### 3.2 Clinical landscape of CD19 CAR-T therapy for LBCL

In R/R LBCL, CD19 CAR-T therapy has demonstrated consistent and sustained response rates in the second and third line or beyond settings (5–7, 13, 54). Outcome measures of CAR-T efficacy reported in studies include overall response rate (ORR), complete response (CR), duration of response (DOR), PFS and OS. Tisa-cel, axi-cel and liso-cel were initially investigated in single arm trials in R/R LBCL after two or more lines of treatment. In the ZUMA-1 trial, axi-cel showed an ORR of 82% with a CR rate of 54% and median PFS and OS of 5.9 and 25.8 months respectively (5, 55). In the JULIET trial, tisa-cel demonstrated an ORR of 52% and CR rate of 40%, with median PFS of 2.9 months and OS of 11.1 months (6, 56). The TRANSCEND NHL001 study of liso-cel showed an ORR of 73% and CR rate of 53%, with a median PFS and OS of 6.8 and 27.3 months respectively (13, 57).

In the second line setting for R/R LBCL, randomized phase 3 multicenter clinical trials demonstrated the superiority of axi-cel and liso-cel over standard of care (SOC) chemotherapy followed by ASCT for suitable responders. The ZUMA-7 trial showed higher ORR and CR rates with axi-cel than SOC (83% vs 45% and 65% vs 32% respectively), with improved median PFS (14.7 vs 3.7 months) and unreached median OS despite 56% of SOC patients receiving subsequent cellular therapy (7, 58). Similarly, in the TRANSFORM trial liso-cel showed superior ORR (87% vs 49%) and CR rate (74% vs 43%) compared with SOC, with unreached median PFS and OS versus 6.2 and 29.9 months respectively (54). In contrast, tisa-cel did not demonstrate improved EFS over SOC (59). Liso-cel and axi-cel are FDA approved for third or greater line LBCL and for second line patients that are refractory to first line chemoimmunotherapy or have relapsed within 12 months, including patients ineligible for ASCT (18, 60).

### 3.3 Clinical factors associated with efficacy of CD19 CAR-T in LBCL

Pre-treatment clinical features have been associated with an inferior response to CD19 CAR-T therapy. Key factors that associate with poor efficacy include high tumor burden, high lactate dehydrogenase, extranodal disease, and composite risk scores, such as a high International Prognostic Index (IPI) (20, 21). Age has been variably associated with efficacy of CD19 CAR-Ts, with a subset of studies showing either increased or decreased risk with advanced age (61–63). Furthermore, distinct LBCL entities may differ in their response to CAR-Ts (**Table 1**, **2**). An inflammatory state, identified by pre-infusion laboratory biomarkers such as high C-reactive protein (CRP), low albumin, and high ferritin, associates with inferior efficacy, as does poor performance status (20–22, 74, 75).

**Table 1:** Outcome correlates by LBCL histological subtype and cell-of-origin in pivotal CAR-T trials.

Trial	CAR-T product and therapy line	Trial LBCL inclusion criteria and patient population	Clinical outcomes	Cell-of-origin outcomes	LBCL subtype outcomes
Neelapu et al NEJM 2017 (5) Neelapu et al Blood 2023 (55)	Axi-cel >2L	DLBCL, tFL, PMBCL 101 pts received CAR-T Updated 5Y analysis at median FU 63.1m	ORR 83% CRR 58% Median PFS 5.9m Median EFS 5.7m Median OS 25.8m Median DOR 11.1m	No impact of COO subtype on outcome	Best ORR similar for DLBCL vs PMBCL/tFL subgroups (82% vs 83%)
Schuster et al NEJM 2018 (6) Schuster et al Lancet Oncol 2021 (56)	Tisa-cel >2L	DLBCL, tFL, HGBL, LBCL with MYC and BCL2 and/or BCL6 rearrangements 115 pts received tisa-cel Updated analysis at median FU 40.3m	ORR 53% CRR 39% Median PFS 2.9m Median EFS 2.8m Median OS 11.1m	Similar ORR for GCB and ABC subtypes	Better 24m PFS in tFL vs DLBCL subgroup
Abramson et al Lancet 2020 (13) Abramson et al Blood 2024 (57)	Liso-cel >2L	DLBCL, HGBL, PMBCL, G3B FL, tDLBCL, LBCL with MYC and BCL2 and/or BCL6 rearrangements 270 pts received liso-cel Updated 2Y FU (257 pts analyzed)	ORR 73% CRR 53% Median PFS 6.8m Median OS 27.3m Median DOR 23.1m	Not reported	PMBCL and tFL pts had improved PFS and DOR
Locke et al NEJM 2021 (7) Westin et al NEJM 2023 (58)	Axi-cel 2L vs ASCT	RR LBCL within 12m of 1L CIT - DLBCL, tFL, HGBL, LBCL with MYC and BCL2 and/or BCL6 rearrangements, THRBCL, primary cutaneous DLBCL-leg type, EBV-positive DLBCL SOC arm (179 pts): 2–3 cycles platinum-based CIT + ASCT (if CR/PR achieved) Axi-cel arm: 180pts Median FU 47.2m (359 pts)	Axi-cel vs SOC: ORR: 83% vs 50% CRR: 65% vs 32% Median PFS: 14.7m vs 3.7m Median EFS: 10.8m vs 2.3m Median OS: NR vs 31.1m	HR for event/death favoring axi-cel arm for both GCB and ABC subtypes	ORR similar between histological subtypes in axi-cel arm
Bishop et al NEJM 2021 (59)	Tisa-cel 2L vs ASCT	RR LBCL within 12m of 1L CIT – DLBCL NOS, tDLBCL, HGBL, PMBCL, G3B FL, LBCL with MYC and BCL2 and/or BCL6 SOC arm (160 pts): investigator’s choice of 4 prespecified platinum-based chemotherapy regimens + HDT-ASCT Tisa-cel arm: 162 pts Median FU 10m	Tisa-cel vs SOC: Best ORR (at/after 12 weeks): 46.3% vs 42.5% CR (at/after 12 weeks): 28.4% vs 27.5% Median EFS: 3m both groups PFS and median OS not reported in analysis	No significant difference in HR for event/death seen by COO subtype	EFS and OS shorter for HGBL pts vs PMBCL or DLBCL in both arms
Kamdar et al Lancet 2022 (8) Abramson et al Blood 2023 (54)	Liso-cel 2L vs ASCT	RR LBCL within 12m of CIT - DLBCL NOS, tDLBCL, G3B FL, HGBL, PMBCL, LBCL with MYC and BCL2 and/or BCL6, THRBCL SOC arm (92 pts): 3 cycles of CIT (R-DHAP, RICE or R-GDP) + ASCT Liso-cel arm: 92 pts Median FU 17.5m	Liso-cel vs SOC: ORR: 87% vs 49% CR: 74% vs 43% Median PFS: NR vs 6.2m Median EFS: NR vs 2.4m Median OS: NR vs 29.9m Median DOR: NR vs 9.3m	Similar EFS between COO subtypes	EFS similar between DLBCL and HGBL subgroups

2L, Second Line; ABC, Activated B-cell type; ASCT, Autologous Stem Cell Transplant; CIT, Chemoimmunotherapy; CR, Complete Response; CRR, Complete Response Rate; DLBCL, Diffuse Large B-cell Lymphoma; DLBCL NOS, Diffuse Large B-cell Lymphoma, Not Otherwise Specified; DOR, Duration of Response; EFS, Event-Free Survival; FU, Follow-up; GCB, Germinal Centre B-cell type; HGBL, High-Grade B-cell Lymphoma; HR, Hazard Ratio; LBCL with MYC and BCL2 and/or BCL6, Large B-cell Lymphoma with MYC and BCL2 and/or BCL6 rearrangement; NR, Not reached; ORR, Overall Response Rate; OS, Overall Survival; PFS, Progression-Free Survival; PMBCL, Primary Mediastinal B-cell Lymphoma; PR, Partial Response; R-DHAP, Rituximab, Dexamethasone, Cytarabine, Cisplatin; RICE, Rituximab, Ifosfamide, Carboplatin, Etoposide; R-GDP, Rituximab, Gemcitabine, Dexamethasone, Cisplatin; SOC, Standard of Care; tDLBCL, Transformed Diffuse Large B-cell Lymphoma; tFL, Transformed Follicular Lymphoma; THRLBCL, T-cell/Histiocyte-rich Large B-cell Lymphoma.

**Table 2:** Outcome correlates by histological LBCL subtype in real world CAR-T registry analyses.

Trial	CAR-T product and therapy line	LBCL subtype and patient population	Clinical outcomes
Thiruvengadam et al Am J Hematol 2025 (64)	Axi-cel, tisa-cel or liso-cel ≥2L	tiNHL (tFL, tMZL, tWM) 338 tiNHL pts and 844 dnLBCL pts (DLBCL, HGBL)	tiNHL vs LBCL: 2Y PFS: 40.6% vs 38% 2Y OS: 57.6% vs 52% Best ORR: 83% vs 81% CRR: 67% vs 59%
Thiruvengadam et al Transplantation and Cellular Therapy (Abstract) 2025 (65)	Commercial CAR-T ≥2L	tFL 923 pts received CAR-T CIBMTR	2Y PFS: 43% 2Y OS: 57% ORR: 76% CRR: 63%
Nadiminti Transplantation and Cellular Therapy 2025 (66)	Axi-cel, tisa-cel or liso-cel ≥2L	Richter transformation 140 pts received CAR-T CIBMTR	2Y PFS: 32.5% 2Y OS: 46.6% ORR: 71% CRR: 57%
Gauthier et al Am J Hematol 2025 (67)	Axi-cel or tisa-cel ≥2L	PMBCL 135 pts received CAR-T CIBMTR	2Y PFS: 58.6% 2Y OS: 80.8% Best ORR: 79% CRR: 67.7%
Pophali et al Blood Adv 2024 (68)	Axi-cel, tisa-cel or liso-cel ≥2L	THRLBCL pts 58 pts received CAR-T CIBMTR	2Y PFS: 29% 2Y OS: 42% D100 ORR: 43% D100 CRR: 24%
Hossain et al Transplantation and Cellular Therapy (Abstract) 2025 (69)	Axi-cel, tisa-cel or liso-cel ≥2L	HGBL-NOS 111 pts received CAR-T CIBMTR	2Y PFS: 28.7% 2Y OS: 41.6% ORR: 67.6% CRR: 51.4%
Stephan et al Blood Adv 2025 (70)	Axi-cel or tisa-cel >2L	tiNHL (tFL, tMZL) 85 tiNHL pts and 85 dnLBCL pts received CAR-T DESCAR-T	tiNHL vs dnLBCL: 1Y PFS: 55.8% vs 31.7% 1Y OS: 72.1% vs 50.7% ORR: 82.4% vs 63.5% CRR: 63.5% vs 50.6%
Bensaber et al BJHaem 2025 (71)	Axi-cel or tisa-cel ≥1L	Richter transformation 15 pts received CAR-T DESCAR-T	1Y PFS: 48.9% 1Y OS: 46.6% Best ORR: 53.3% Best CRR: 46.7%
Galtier et al HemaSphere 2025 (72)	Axi-cel, tisa-cel or liso-cel ≥2L	PMBCL 82 pts received CAR-T DESCAR-T	2Y PFS: 57.4% 2Y OS: 73.8% Best ORR: 84.7% 3M CRR: 51.5%
Phina-Zieben et al Blood Adv 2025 (73)	Axi-cel or tisa-cel >2L	HGBL 60 HGBL pts and 135 LBCL (dnLBCL, tFL, tMZL) pts received CAR-T DESCAR-T	HGBL vs non-HGBL: Median PFS: 3.2m vs 4.5m Median OS: 15.4m vs 18.3m ORR: 68% vs 76% CRR: 60% vs 59%

2L, Second Line; AE, Adverse Event; CIBMTR, Center for International Blood and Marrow Transplant Research; CRR, Complete Response Rate; CRS, Cytokine Release Syndrome; DESCAR-T, Dispositif d’Enregistrement et Suivi des patients traités par CAR-T cells (French Nationwide Registry for Patients Treated by CAR-T cells); DLBCL, Diffuse Large B-cell Lymphoma; dnLBCL, *de novo* Large B-cell Lymphoma; HGBL, High-Grade B-cell Lymphoma; HGBL-NOS, High-Grade B-cell Lymphoma, Not Otherwise Specified; ICANS, Immune Effector Cell-Associated Neurotoxicity Syndrome; LBCL, Large B-cell Lymphoma; ORR, Overall Response Rate; OS, Overall Survival; PFS, Progression-Free Survival; PMBCL, Primary Mediastinal B-cell Lymphoma; tFL, Transformed Follicular Lymphoma; THRLBCL, T-cell/Histiocyte-rich Large B-cell Lymphoma; tiNHL, Transformed Indolent Non-Hodgkin Lymphoma; tMZL, Transformed Marginal Zone Lymphoma; tWM, Transformed Waldenström Macroglobulinemia.

The quality of the LD regimen is an important factor. The best outcomes have been associated with cyclophosphamide and fludarabine (CyFlu) lymphodepletion or bendamustine-based lymphodepletion (15, 76, 77). Higher intensity of CyFlu resulting in an increase in T-cell homeostatic cytokines after LD has been associated with better efficacy (15, 74).

The selection and characteristics of the CAR-T infusion product can influence efficacy. While axi-cel and liso-cel reportedly demonstrate equivalent survival outcomes, tisa-cel appears to be associated with inferior efficacy in LBCL (78, 79). Infused products comprising a higher fraction of less-differentiated T cell phenotypes have been associated with better efficacy (22, 23, 58, 80, 81). Peak *in vivo* CAR-T counts and the area under the curve of CAR-T counts in the first 28 days after infusion (AUC_0-28_) correlate with treatment response in some studies (5, 7, 13, 15, 22, 55, 59, 81). However, in other studies no association was observed, suggesting that other factors such as CAR-T dysfunction in the lymphoma TME may contribute (8, 56, 58, 82).

Early treatment response by fludeoxyglucose‐18 positron emission tomography with computed tomography (18‐FDG PET/CT) and circulating tumor DNA dynamics at certain time points associate with durable survival, although further consensus on the optimal timing of testing and method of circulating tumor DNA assessment is required (83, 84).

Despite the presence of many factors that associate with inferior efficacy of CD19 CAR-Ts, prediction of the outcome in each patient is not yet possible, suggesting that other considerations are involved. The TME is a critical and often underappreciated factor that may govern efficacy outcomes. This review will focus on elements of the LBCL TME that associate with response to CD19 CAR-T therapy.

## 4 Histological and cellular correlates of CAR-T response in LBCL

### 4.1 Association of defined LBCL histologic and cell-of-origin subclassifications with CAR-T efficacy

Based on characteristics of the tumor, LBCL is subclassified into histological and cell-of-origin subtypes. Numerous studies have assessed the prognostic relevance of these subtypes in the context of CD19 CAR-T therapy. Although, as described earlier, WHO-HAEM5 represents the contemporary classification system, much of the existing CAR-T literature reports outcomes based on the preceding WHO-HAEM4 histologic categories.

#### 4.1.1 Histological subtypes of LBCL

**Figure 2:**
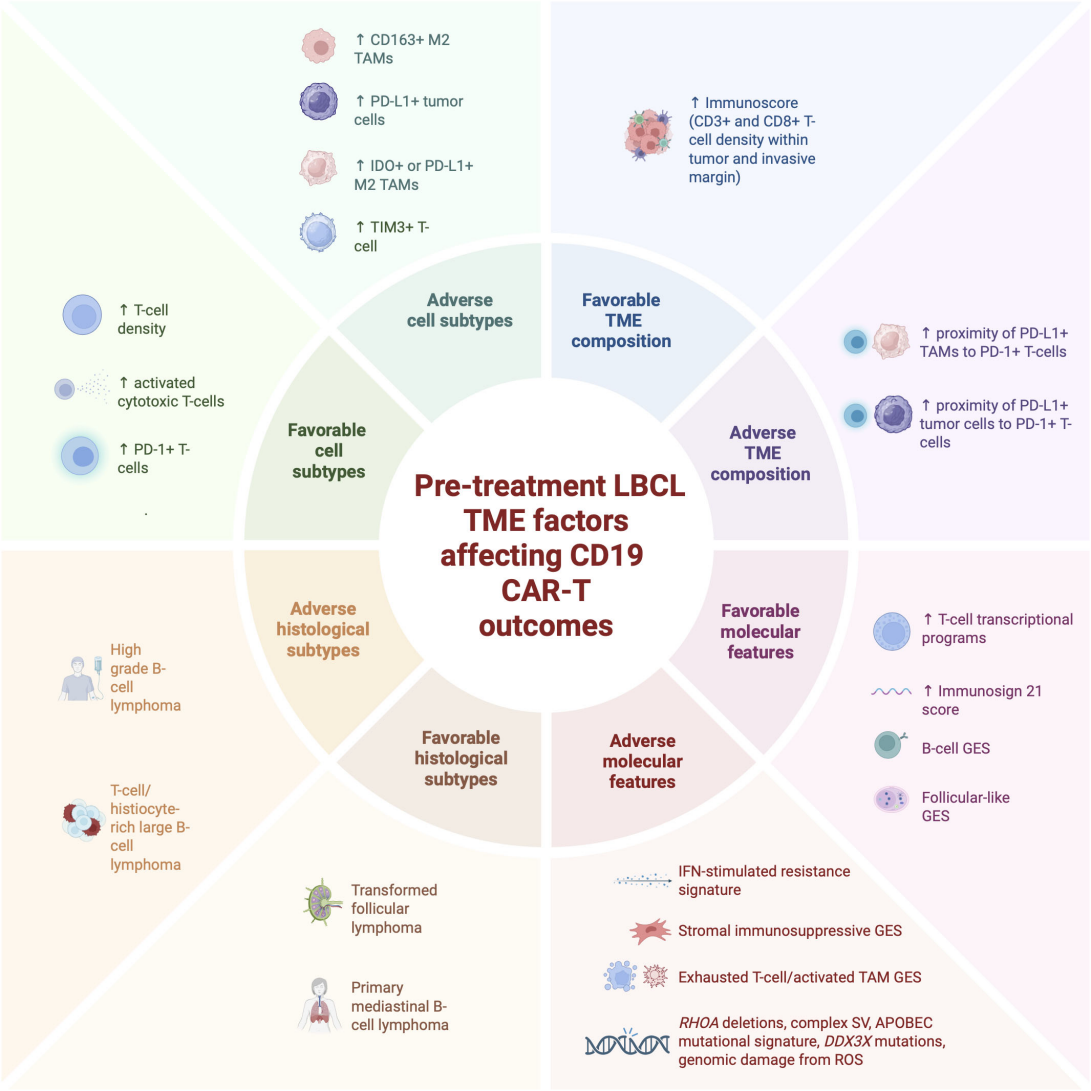
Pre-treatment LBCL TME factors affecting CD19 CAR-T outcomes. Overview of the pre-treatment LBCL tumor microenvironment features associated with favorable or adverse clinical responses to CD19 CAR-T therapy. The diagram summarizes key categories of tumor microenvironment determinants, including cellular composition, histological subtypes, and molecular transcriptional programs, and highlights representative factors linked to improved or poor outcomes. Favorable features include increased T cell density, activated cytotoxic T cells, histological subtypes such as transformed follicular lymphoma and primary mediastinal B-cell lymphoma, as well as transcriptional programs indicative of robust immune activation. Adverse features include enrichment of suppressive myeloid and regulatory cell subsets, close proximity of PD-L1^+^ tumor cells or TAMs to T cells, unfavorable histology (e.g., high-grade B-cell lymphoma and T-cell/histiocyte rich large B-cell lymphoma), and molecular programs associated with immune dysfunction or resistance. LBCL, large B-cell lymphoma; TME, tumor microenvironment; MDSC, myeloid-derived suppressor cell; TAN, tumor-associated neutrophil; TAM, tumor-associated macrophage; GES, gene expression signature.

Response to CD19-directed CAR-T therapy may be affected by the histological subtype of LBCL (**Figure 2**). Distinct histological subtypes are characterized by differences in the TME, including variation in cellular subtype proportions, immune composition, and associated molecular signatures and key signaling pathways, all of which may influence CAR-T efficacy. In the initial ZUMA-1 and JULIET trials, histological subtypes did not associate with responses to CAR-T therapy; however, updated analysis of the JULIET trial showed longer PFS at 24 months in transformed follicular lymphoma (tFL) patients (**Table 1**) (5, 6, 56). In the TRANSCEND trial, the DOR and PFS were longer in patients with PMBCL and tFL compared to other LBCL subtypes (13). Subgroup analysis of the BELINDA trial showed HGBL patients demonstrated inferior EFS and OS compared to DLBCL and PMBCL patients in both the tisa-cel and SOC groups. In contrast, other second line LBCL CAR-T trials reported no outcome difference between histological subtypes (8, 58, 59, 82). The relatively small numbers of patients treated in these studies may have limited the power to identify differences in response between histologies.

Multiple recent large retrospective “real-world” studies have examined datasets reporting outcomes in different LBCL subtypes following CAR-Ts (**Table 2**). While limited by their retrospective nature, these studies have been helpful in identifying potential differences in susceptibility of different LBCL histologies to CAR-Ts. In data from the Center for International Blood and Marrow Transplant Research registry, PMBCL patients demonstrated the most favorable outcomes with a CRR of 67.7% and 2 year PFS of 58.6%, followed by tFL with a CRR of 63% and 2 year PFS of 43% (65, 67). Conversely, THRLBCL demonstrated the worst outcomes with a Day 100 CRR of 24% and 2 year PFS of 29% (68). HGBL-NOS and Richter transformation (RT) patients demonstrated good CR rates albeit with low PFS rates, with CRRs of 51.4% and 57%, and 2 year PFS rates of 28.7% and 32.5%, respectively (66, 69).

In the French DESCAR-T registry studies, patients with tiNHL, including tFL and transformed marginal zone lymphoma (tMZL) histologies, had better outcomes compared to *de novo* DLBCL patients with a 1 year PFS of 55.8% vs 31.7% and CRRs of 63.5% vs 50.6% (70). PMBCL patients also demonstrated favorable outcomes with a 2 year PFS of 57.4% and 3 month CRR of 51.5% (72). HGBL patients had similar outcomes to a matched non-HGBL group in terms of OS (3.2m vs 4.5m) and CRR (60% vs 59%) (73). A small DESCAR-T registry study of 15 RT patients demonstrated reasonable response rates with a best CRR of 46.7% and 1 year PFS of 48.9%; however AEs rates were higher than expected with grade >3 CRS and ICANS rates of 40% and 33%, respectively (71). A large real-world UK study comparing outcomes of LBCL subtypes in 760 axi-cel or tisa-cel treated patients reported the best survival outcomes in tFL and PMBCL, with 12 month OS rates of 84% and 58% respectively (85). A US multi-center study demonstrated superior CRRs in tiNHL patients compared to aggressive LBCL patients, including dnDLBCL and HGBL, at 67% vs 59% respectively (64). Despite the inherent limitations of retrospective, non-randomized real world CAR-T registry studies, the overall findings suggest that tFL and PMBCL histologies may exhibit comparatively better therapeutic outcomes following CD19 CAR-T therapy.

#### 4.1.2 Cell-of-origin molecular subtypes of DLBCL

Early gene expression profiling studies described molecular COO DLBCL subgroups with prognostic significance following anthracycline-containing first line chemotherapy regimens (86, 87). These two biologically distinct COO subgroups reflect tumors with GESs reminiscent of normal germinal center B-cells (GCB subtype) or of activated B-cells (ABC subtype). These molecular subtypes, arising from B-cells at different stages of maturation, underscore how COO-associated transcriptional programs intersect with the TME to influence prognostic outcomes. Several molecular predictive scores and immunohistochemical assays have been developed for accurate case assignment (88–93). The dark zone signature, also known as the double-hit/molecular high-grade signature, was later described, identifying a discrete high-risk subgroup of GCB-type DLBCL patients (92, 94).

Although DLBCL cases assigned to the ABC/non-GCB molecular subtype were previously shown to display inferior OS and EFS following first line treatment with multiagent anthracycline-based chemotherapy, this was not the case in pivotal trials of CD19 CAR-Ts in R/R LBCL. In the third line or later DLBCL setting, COO subtypes demonstrated similar outcomes in the ZUMA-1 and JULIET trials; analysis of outcomes by COO subgroup was not reported in the TRANSCEND trial (5, 6, 13). In the second line setting, there was no difference in treatment outcomes between GCB and ABC COO subgroups following axi-cel, liso-cel or tisa-cel treatment (7, 8, 59). Differences in response by COO subgroup have also not been reported in other studies (95, 96).

### 4.2 Associations of distinct cell subsets in the TME with efficacy of CAR-Ts

#### 4.2.1 Associations of tumor cell phenotype with CAR-T efficacy

The impact of pre-immunotherapy tumor CD19 expression on clinical outcomes has been examined in multiple studies. In the ZUMA-1 and JULIET trials, pre-treatment CD19 protein expression level by immunohistochemistry (IHC) or immunofluorescence did not associate with response (5, 6). In the analysis of axi-cel treated patients on the ZUMA-7 trial, high CD19 gene and protein expression was associated with longer EFS. Low tumor CD19 expression combined with higher CCR7+/CD45RA+ T-cells in the final infusion product was also associated with longer EFS; however, patients with low CD19 protein expression with an immunosuppressive stromal gene expression pattern displayed shorter EFS (97). Another study reported no association between *CD19* genetic aberrations, CD19 expression by flow cytometry or by transcriptional profiling and outcome in axi-cel treated patients (98).

There does not appear to be a consistent and uniform relationship between CD19 expression and CD19 CAR-T outcome, although loss of CD19 expression is a plausible mechanism of treatment failure (99). While it is highly likely that target antigen expression is needed, there are factors that render the identified associations between CD19 expression and outcomes of CD19 CAR-Ts inconsistent, including sensitivity constraints of the commonly used immunohistochemical and flow cytometric assays, small patient sample sizes in published correlative studies, and limitations of testing a single lesion to determine overall antigen expression levels. Complete responses can still be achieved despite the presence of CD19-negative tumor populations, suggesting CAR-independent mechanisms of killing. When CD19 is undetectable by both IHC and qPCR, higher sensitivity assays that improve the quantitation of low-level pre-treatment CD19 expression might better identify patients at risk of inferior PFS following CD19 CAR-T (100). A higher percentage of malignant B-cells positive for PD-L1 and MHC-II have also been described in non-durable responders following CAR-T therapy (101). A lower density of PD-L1+ tumor cells in pre-LD LBCL biopsies has been described in CR patients (102). Aberrations in CD58 on LBCL tumors have also been associated with inferior CD19 CAR-T efficacy compared to patients without CD58 alterations (103).

#### 4.2.2 Associations of immune cell subtypes with CAR-T efficacy

The immune composition of the TME plays a key role in regulating tumor recognition and immune rejection; and T-cells are a cornerstone of the anti-tumor immune response. Subsets of T-cells in the TME display different levels of maturation, activation and exhaustion. In DLBCL, a GEP consistent with enrichment of CD8+ T cells with low effector molecules and high inhibitory receptors (referred to as “terminally exhausted”) associated with inferior survival (104). In liso-cel treated patients, higher PD-1+ T-cells in pre-treatment biopsies associated with CR at one month (95). In this study, post-treatment biopsies of patients in CR at 3 months showed higher CD4+ and CD8+ CAR-Ts and non-CAR-Ts. A majority of T cells in the TME early after CAR-T infusion were not identified as CAR-Ts (which only represented ~5% of total T-cells at Day 11 post infusion) (105). In another study including 17 post-treatment patients, CAR-Ts also comprised a minority of T-cells within the tumor after axi-cel infusion, with <5% of intratumoral T-cells found to express a CAR by immunofluorescence at 5 or more days following infusion. The remaining T-cells that did not express a CAR displayed an activated phenotype (105). Future studies may determine if these CAR-negative T-cells are CAR-engineered T cells that don’t express the CAR or if they are endogenous T-cells. CAR-Ts may not act alone; their initiation of an antigen-directed anti-tumor immune response may enable subsequent infiltration of endogenous T-cells (105). However, the extent of the contributions from T-cells that do or do not express a CAR remains unclear.

In a study by Jin et al, a higher fraction of CD4+ T cells in biopsies at relapse was associated with longer remission duration, whereas patients with PD had higher proportions of pre-treatment NK cells and fibroblasts (106). Poorer responses, including PD or shorter remission duration, were associated with higher TIM-3 expression on CD8+ and CD4+ T-cells, higher T-cell Ki67 and CD57 expression, and lower CD69 expression. Pre-treatment T-cell subset fractions were similar between CR and PD patients, although fewer functional T-cells were seen in poor response patients. A TME enriched with CD8+ T cells rather than tumor cells was associated with longer remission. In a small phase 1 trial of 10 B-NHL patients receiving CD19 CAR-T therapy, day 11 biopsies showed T-cells were distributed within the tumor in patients who subsequently achieved CR, with fewer B-cells and macrophages; whereas patients who subsequently achieved PR had a higher proportion of macrophages with fewer CD3+ T-cells (107). CR patients had higher levels of both endogenous T-cells and CAR-Ts in post-treatment biopsies with fewer TAMs by confocal microscopy. In the JULIET trial, there was no difference in outcome based on the percentage of total cells or CD3+ T-cells expressing immune checkpoint proteins in pre-treatment biopsies (PD-1, PD-L1, TIM-3, LAG-3) (6).

The Immunoscore is an immunohistochemical method of assessing cytotoxic T-cell infiltration within the tumor core and invasive margin that has shown prognostic utility in solid organ malignancy (108). Scholler et al. performed multiplex IHC on pre- and post-treatment biopsies from axi-cel treated LBCL patients, focusing on the influence of the spatial distribution of immune cell types including Immunoscore on clinical outcome (109). In this study, various immunohistochemical combinations representing infiltration of cytotoxic T-cells, exhausted cytotoxic T-cells and suppressive cells were investigated and the response to axi-cel trended positively with a higher Immunoscore. The Immunoscore demonstrated a positive association with OS. In 10 paired samples, composite assessments comprising lower tumor burden by sum of product diameters with higher T-cell density by IHC in pre- and post-treatment samples were associated with achievement of CR. Activated cytotoxic T-cell density, as determined by the density of CD8+ T-cells expressing PD-1 with or without LAG-3 in the absence of TIM-3, was associated with OS. Higher peak CAR-T levels were associated with a lower density of exhausted TME T-cells, without expression of PD-1, LAG-3, TIM-3 or TOX.

TAM subsets are well described in cancer as having pro-tumor qualities. Co-culturing of CAR-Ts and Raji tumor cells with M2 macrophages *in vitro* results in reduced CAR-T cytotoxic activity, proliferation, and cytokine secretion (110). In patients receiving a phase 1 4-1BB-costimulated CD19 CAR-T product, increased pre and post-treatment M2 TAMs were noted in patients who did not achieve a CR; and co-culturing of M2 TAMs with T-cells suppressed CD4+ and CD8+ T-cell proliferation *in vitro* (107). In liso-cel treated patients, higher CD163+/IDO1+ macrophages, CD163+/PD-L1+ macrophages, and proliferating tumor cells were observed in the TME of patients who subsequently developed progressive disease (95). In an analysis of pre-LD LBCL biopsies, lower densities of PD-L1 expressing macrophages and tumor cells and higher density of CD4+ T cells were seen in CR patients (102).

MDSCs are immature myeloid cells of granulocytic or monocytic origin that can dampen the tumor immune response (26). *In vitro* studies have shown suppression of CAR-T transduction efficiency, activation, proliferation and function when co-cultured with MDSCs during T-cell transduction and stimulation (111). High levels of baseline peripheral blood monocytic-MDSCs are also associated with low CAR-T expansion and short durability of response to axi-cel (101).

#### 4.2.3 Immune cell interactions and cross-talk within the LBCL TME following CD19 CAR-Ts

**Figure 3:**
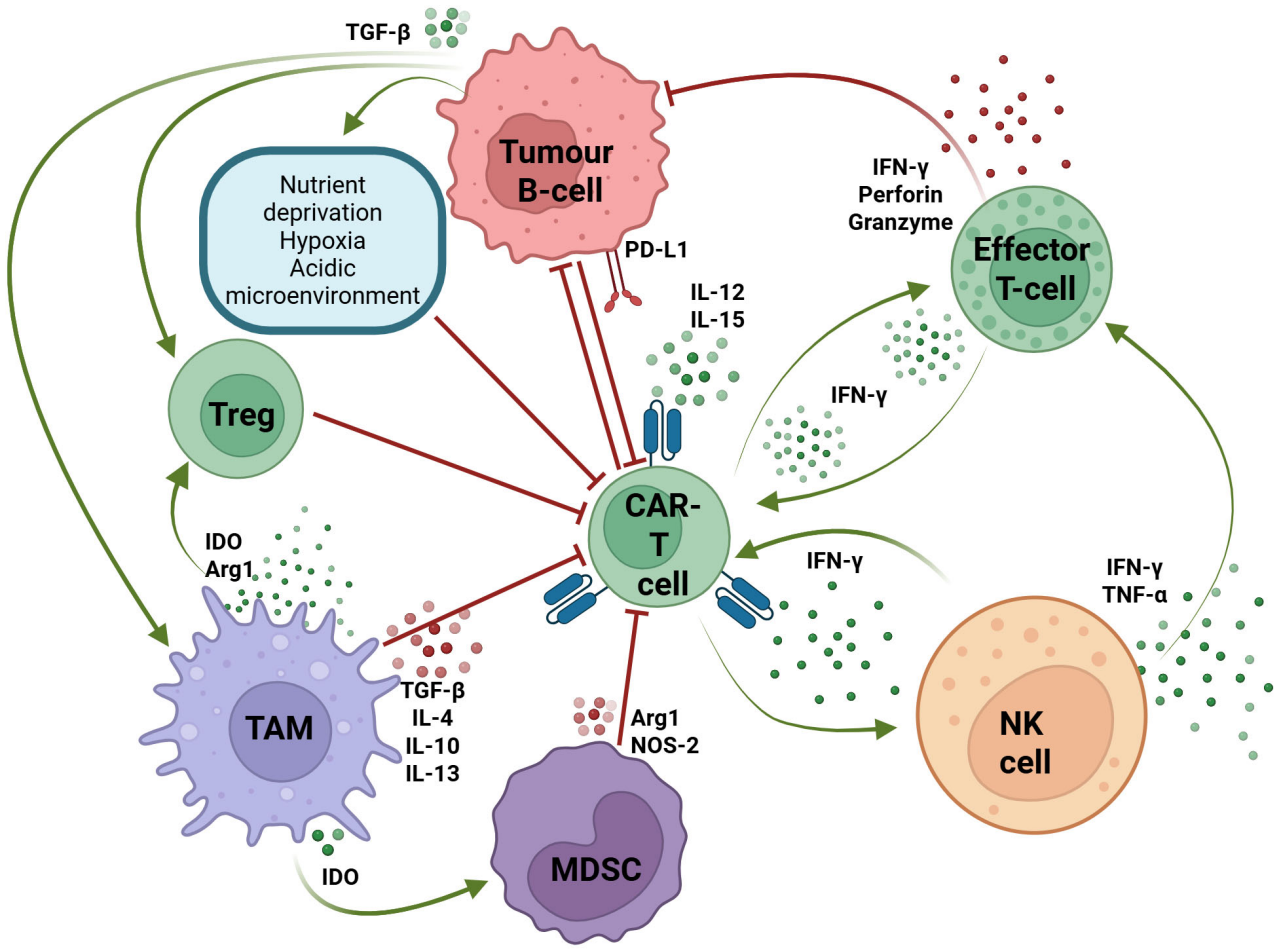
Stimulatory and immunosuppressive interactions within the LBCL TME shaping CD19 CAR-T function. Following infusion, CAR-Ts localize to the tumor and secrete IFN-γ, promoting recruitment and activation of local immune cells. As shown on the right-hand side of the figure, NK cells produce IFN-γ and TNF-α, which can enhance endogenous effector T-cell responses. Effector T-cells further amplify anti-tumor immunity through IFN-γ secretion and direct tumor cell killing via perforin and granzyme release. Stimulatory cytokines within the TME, including IL-12 and IL-15, support CAR-T proliferation, persistence, and effector function. Concurrently, multiple immunosuppressive components of the TME inhibit CAR-T activity, as depicted on the left-hand side of the figure. TAMs express Arg1 and IDO, promoting recruitment of Tregs and MDSCs, which suppress CAR-T function, while MDSCs further mediate suppression via Arg1 and NOS2. Tumor cells additionally impose metabolic and physical stress through nutrient deprivation, hypoxia, and an acidic microenvironment, and express PD-L1 to inhibit T-cell activity, while immunosuppressive cytokines (TGF-β, IL-10, IL-4, IL-13) limit CAR-T infiltration, activation, and effector capacity. LBCL, large B-cell lymphoma; IFN-γ, interferon-γ; natural killer cell, NK cell; TNF-α, tumor necrosis factor-α; IL-12, interleukin-12; IL-15, interleukin-15; TAM, tumor-associated macrophage; Arg1, arginase-1; IDO, indoleamine 2,3-dioxygenase; Treg, regulatory T cell; MDSC, myeloid-derived suppressor cell; VEGF, vascular endothelial growth factor. Created in BioRender. Turtle, C. (2026) https://BioRender.com/x0kekk1.

The LBCL TME is not a static, unidirectional system but instead comprises dynamic and reciprocal interactions between CAR-Ts and resident tumor and immune components that together shape the magnitude and durability of anti-tumor responses (**Figure 3**). Sustained cytotoxic activity appears to depend on active cellular cross-talk between CAR-T subsets and the TME. Using an immunocompetent syngeneic mouse model of Burkitt-like lymphoma, Boulch et al. demonstrated that effective anti-tumor responses require coordinated intra-tumoral interactions, with infused CD8^+^ CAR-Ts mediating direct tumor cell killing and CD4^+^ CAR-Ts primarily driving activation of the endogenous immune compartment (112). IFN-γ release by CAR-Ts promoted local immune cell recruitment and activation, inducing secondary IL-12 production that further amplified the anti-tumor response. Complementary findings by Cazaux et al. reported rapid CAR-T-mediated tumor cell killing but observed site-specific differences in cytotoxic efficacy between lymph node and bone marrow niches, highlighting how regional variation in TME-mediated cellular cross-talk and immunosuppression influences CAR-T function (113). While extensive work has characterized dynamic cell-cell interactions within the LBCL TME (see Section 2: The immune composition of the LBCL TME), comparatively little is known about direct cellular interactions involving infused CD19 CAR-Ts within the LBCL TME following therapy. Evidence for CAR-T-TME cross-talk has largely been inferred from post-infusion single-cell transcriptomic analyses, bulk gene-expression signatures or *ex vivo* functional assays rather than direct *in situ* observation. Consequently, the spatial and cellular contexts in which CAR-Ts engage with immune, stromal and myeloid elements in the LBCL TME remain poorly defined. Future spatially resolved and *in situ* studies will be critical to delineate CAR-T-TME interaction patterns that may correlate with treatment efficacy or resistance.

## 5 Molecular determinants of CAR-T response in LBCL

### 5.1 Association of genetic DLBCL subtypes with CAR-T efficacy

DLBCL has also been subcategorized into genetic subtypes that associate with prognosis based on the presence of genomic aberrations such as mutations, translocations and copy-number alterations, including the LymphGen and Chapuy classifiers (114–116). Distinct genetic subtypes correlate with inferior outcomes in newly diagnosed DLBCL including the MCD and N1 LymphGen subgroups, defined by *MYD88^L265P^* and *CD79B* mutations, and *NOTCH1* mutations, respectively (115, 116). Chapuy subgroups demonstrating worse prognosis include the C3 subgroup, enriched with *EZH2*, *KMT2D* and *CREBBP* mutations and *BCL2* rearrangements, and the C5 subgroup, characterized by *MYD88^L265P^* and *CD79B* mutations with 18q gains (114). In a study of 49 RR LBCL patients by Jain et al, CAR-T outcome did not correlate with Chapuy or LymphGen classification group, or the presence of double hit (MYC and BCL2 and/or BCL6 rearrangements) or double expressor status (98). Sworder et al. also found no difference in response to CAR-T therapy by LymphGen subtype in a study of 138 patients, although R/R LBCL patients pre-CAR-T had a higher prevalence of GCB COO and EZB (*EZH2* mutations and *BCL2* translocations) or A53 (aneuploidy with *TP53* inactivation) LymphGen genetic subtypes than treatment naïve DLBCL patients (96). Shi et al. also found no difference in CAR-T outcome between genetic clusters resembling LymphGen subtypes in a study of 84 RR LBCL patients (117). Although Hill et al. did not demonstrate statistically significant differences between subgroups in outcomes in a study of 96 R/R LBCL patients pre-CAR-T, a trend toward inferior PFS in the LymphGen EZB subgroup and improved outcomes in the MCD and A53 subgroups were reported (118). Genetic data for each subgroup was not reported in the pivotal CD19 CAR-T trials. The genetic subtype of DLBCL may therefore have better predictive value at initial diagnosis compared to the context of CAR-T therapy.

### 5.2 Association of genetic aberrations with CAR-T efficacy

Well described adverse genetic aberrations, including specific gene mutations and structural variants at initial LBCL diagnosis, have not consistently associated with outcomes in the CAR-T setting. While *TP53* alterations may associate with poorer outcomes following CAR-T, the current evidence is not definitive. Studies have demonstrated inferior outcomes following CAR-T therapy in the presence of *TP53* alterations, including inferior EFS, OS and CR rates (96, 117, 119). However, this association was not confirmed in a study of 49 patients by Jain et al. (98) The effect of *TP53* mutations on OS and PFS was more notable following treatment with 4-1BB costimulatory domain CAR-T products (liso-cel and tisa-cel) compared to axi-cel in one study (119). In this study *TP53* alterations in newly diagnosed DLBCL associated with changes to the TME affecting IFN signaling pathways, expression of certain cytotoxic genes, CD8+ T-cell composition and extrinsic apoptosis pathways. The effect of *TP53* aberrations on CAR-T outcome requires further exploration.

Sworder et al. undertook a multifaceted approach to the analysis of factors leading to axi-cel resistance, simultaneously examining levels of ctDNA, cell-free CD19 CAR-T fragments and cell-free T-cell receptor rearrangements through prospective deep sequencing (96). Although broadly similar, some differences were noted in genomic mutations in LBCL patients at diagnosis compared to those with R/R disease before CAR-T, such as more frequent *TP53*, *MYC*, and *EP300* aberrations and fewer *TOX*, *BCL6* and *CD79B* aberrations in R/R patients. *TMEM30A*, *IRF8*, *PAX5*, *TP53*, *DTX1* and *P2RY8* mutations were associated with shorter EFS following CAR-T therapy. Clonal selection of mutations in *TP53* and *PAX5*, emergent *CD19* mutations and amplification of genomic regions encoding immune checkpoint proteins such as *PD-L1* and *PD-L2* at relapse/progression were also noted. *RHOA* deletions, complex structural variants, certain mutational signatures (APOBEC, genomic damage related to reactive oxygen species) and *DDX3X* mutations have also been found to associate with inferior outcomes (98, 117).

### 5.3 Association of immune-related transcriptional profiles in the TME with CAR-T efficacy

Bulk and single cell RNAseq technologies have been employed to identify cell subsets and gene expression patterns in the TME that associate with outcomes following CD19 CAR-T treatment; immune-related transcriptional programs have been a primary focus. The response to axi-cel can associate with GEP at baseline and following treatment. A transcriptional program suggestive of increased T-cell activity by bulk RNAseq prior to CAR-T infusion and lower expression of MHC class II genes and cancer-testis antigens was associated with better response in the ZUMA-1 trial (120). Axi-cel responders also demonstrated early post-treatment increases by whole tumor bulk RNAseq in expression of genes related to cytotoxic T-cells (*CD8A*, *GZMA*), IFN-mediated immune checkpoint regulation (*CD274*, *CTLA4*, *CD276*), chemokines and myeloid-related genes (*CCL2*, *CD14*, *CD68*) and cytokines (*IL-15*) and reduced expression of B-cell lineage genes post-treatment, suggesting effective malignant B-cell clearance (109). Reduced expression of genes associated with T-cells, immune checkpoints, cytokines, IFN and MHC class I, and increased expression of *CTLA4*, *CCR4* and *CCL22* has been described at relapse. The Immunosign 21 score incorporates a panel of 21 immune-related genes (*CD3D, CD3E, CD3G, CD8A, CD69, ICOS, IL-5, CCL2, CCR2, IRF1, GZMA, GZMB, GZMK, GZMM, PRF1, CXCR3, CXCL10, CXCL11, STAT1, STAT4, TBX21*) reflecting immune response programs; the pre-treatment tumor profile associated with OS following CAR-T therapy (109). In patients who relapse demonstrating high levels of CAR-Ts within the tumor compared to low CAR-T levels, gene set enrichment analysis has shown upregulation of inflammatory response, IFN-γ response, TGF-β signaling, T-cell exhaustion and Treg signatures by bulk RNAseq (96).

IFN signaling is a central driver of anti-tumor immunity, initiating gene expression programs that activate downstream cellular effectors of the immune response. Enrichment of an IFN-stimulated resistance signature (IFN.RS), which correlates with a poorer response to immunotherapy in solid organ malignancy, is also associated with a non-durable response (NDR) to axi-cel (101, 121). In this study, higher expression of IFN signaling targets and macrophage associated genes (*CD163*, *SIGLEC1*) in the pre-treatment LBCL TME by bulk RNAseq testing was seen in the NDR group; poor axi-cel expansion was seen with high expression of IFN signaling genes. Locke et al. categorized the LBCL TME into four subtypes on the basis of pre-treatment immune-related GESs by bulk RNAseq of patients on the ZUMA-7 trial (97). A superior response to axi-cel was associated with a B-cell GES, while inferior outcomes were seen with a stromal immunosuppressive GES. Lower expression of the Immunosign 21, B-cell and T-cell signatures were seen in CAR-T treated and standard of care patients at later lines of therapy compared to the time of initial diagnosis, suggesting changes in the overall tumor immune milieu during the disease trajectory and with subsequent lines of treatment.

The impact of the LBCL TME on liso-cel outcomes was examined by Olson et al, who found superior outcomes were associated with a follicular-like GES, the Immunosign 21 signature and the Lenz stromal-1 and stromal-2 GESs by pre-treatment whole tumor bulk RNAseq (95). At day 11 post infusion, higher T-cell and macrophage-related gene expression, including CAR genes, was associated with CR. Conversely, patients with progressive disease had enrichment of gene sets with a DLBCL-like rather than FL-like GES, *EZH2* targets, transcriptional regulators and cell-cycle pathways.

Progressive disease following an investigational CD19 4-1BB CAR-T product was associated with higher expression of genes by pre-treatment whole tumor bulk RNAseq associated with tumor-associated DCs, tumor-associated fibroblasts, MDSCs and immunosuppressive cytokines, whereas CR patients had reduced expression of negative regulators of TAMs, Treg and MDSC recruitment and upregulation of T-cell activation markers (107). Higher levels of M2 macrophages, downregulation of immune pathways and higher effector T-cell checkpoint expression by scRNAseq in a PD patient compared to a CR patient was reported in one small study (110). In an analysis by Hirayama et al, pre-LD whole tumor bulk transcriptional profiling showed that tumors from patients who achieved CR after CAR-Ts had higher expression of genes involved in T-cell activation, proliferation, trafficking and cytokine production, whereas those who didn’t achieve CR had higher expression of T-cell dysfunction and macrophage-associated genes (102). Of the LymphoMAPs described by Li et al, the subtype enriched with exhausted CD8+ T-cells and activated macrophages showed no improvement in EFS with CAR-T therapy compared with SOC (44).

While the majority of studies interrogating the LBCL TME in the context of CAR-T therapy have utilized bulk RNA-seq approaches, single-cell technologies offer greater resolution for dissecting cell-cell interactions and dynamic cellular processes within the TME. Single cell analyses of PB samples from recipients and CAR-Ts post-infusion have revealed important mechanisms associated with CAR-T efficacy in LBCL. A multimodal single-cell analysis of LBCL patients treated with CD19 CAR-T demonstrated that early post-infusion expansion of polyclonal CAR-Treg cells was associated with inferior clinical responses, potentially through suppression of T-cell proliferation and expansion (122). In the pre-infusion setting, single-cell profiling of PB revealed enrichment of inflammation-driven gene pathways within T-cell and myeloid subsets in CD19 CAR-T non-responders. Conversely, responders showed a high CD4 effector memory/CD4 central memory ratio, enrichment of CD16 monocytes and the presence of both malignant and non-malignant B-cells in PB prior to treatment (123). Accumulating single-cell transcriptomic studies indicate that the efficacy of CD19-directed CAR-T therapy is determined by intrinsic molecular states within infused CAR-Ts, including differentiation status, metabolic fitness, exhaustion signatures, and clonal kinetics, which can be influenced by manufacturing conditions and early *in vivo* expansion (23, 124, 125). However, data describing the molecular states of CAR-Ts directly isolated from within the LBCL TME are currently lacking and therefore the impact of transcriptional heterogeneity on CAR-T function *in situ* remains unclear.

The transcriptional landscape of the LBCL TME undergoes dynamic changes following CAR-T infusion and appears to be associated with the response to CD19 CAR-T therapy. In multiple studies, higher expression of genes associated with T-cells and the Immunosign 21 score correlate with superior outcomes after CAR-T therapy. Given the importance of a robust local T-cell response in mediating the anti-tumor effect within the TME following CAR-T immunotherapy, it stands to reason that GEPs reflecting effective T-cell trafficking to tumor sites and IFN-mediated checkpoint expression suggestive of T-cell activation would be seen in this context. Interpretation of the data from these studies is limited by heterogeneity in study design and laboratory techniques. Going forward, other methods may provide complementary insights to RNAseq, helping to identify potential targetable mechanisms that could enhance aspects of the CAR-T response (126). Developing a deeper understanding of TME GESs that associate with inferior outcomes to CD19 CAR-T therapy has clear implications for determining predictive algorithms of CAR-T response and for the development of targeted therapies.

## 6 Cells in context: association of spatial TME composition with CAR-T efficacy

Spatial imaging technologies highlight the distribution of TME components including tumor cells, immune cells, stromal elements, and tumor vasculature to identify organizational features with potential implications for disease prognosis and treatment response. The ability to examine samples with an increasing number of multiplexed reagents allows for an in-depth analysis of tissue architecture, enabling a focus on immune cell subset composition, distributions and adjacencies. Identification of the spatial conformation of the tumor to generate hypotheses surrounding interactions between networks of immune and other cell components of the TME is now central to enhancing CAR-T therapy for LBCL.

### 6.1 Spatial proteomic analyses of the LBCL TME in CAR-T therapy

Hirayama et al. analyzed the spatial topography of pre-LD LBCL biopsies and found CR patients had higher proportions of hypocellular/fibrotic regions, a lower proportion of interspersed immune infiltrate, and greater T-cell aggregates (102). Close proximity of PD-L1+ macrophages or PD-L1+ tumor cells to PD-1+ T-cells was associated with failure to achieve CR. In a study by Jin et al, analysis of cell-cell interactions demonstrated more fibroblasts and fewer CD4+ T cells in close proximity to tumor cells in poorer response patients (106). In the JULIET trial, no clear effect of the PD-1/PD-L1 interaction score on response was seen, although a small number of patients with the highest PD-1-PD-L1 interaction scores demonstrated poor outcomes (6).

Not only the overall immune composition of the LBCL TME, but also the adjacencies of certain cell types may have prognostic impacts upon response to CAR-T therapy. The presence of a robust overall T-cell infiltrate demonstrating a more activated than dysfunctional state, along with fewer immunosuppressive macrophages demonstrating inhibitory protein expression such as PD-L1 corresponds with better CAR-T outcomes. Conversely, where cell-cell signaling analysis demonstrates the exclusion of T-cells from within the tumor or the colocation of inhibitory PD-L1+ macrophages to anti-tumor PD-1+ T-cells, poorer outcomes are described. The relatively limited number of phenotypic antigens in highly multiplexed IHC assays renders characterization of the interactions between well-defined subsets of immune, stromal and tumor cells difficult. New methods for spatial analyses of single cell transcriptomes in the TME may shed more light on these interactions.

### 6.2 Spatial transcriptomics for TME analysis in lymphoma

Spatial transcriptomics (ST) is an emerging technique for TME assessment that provides simultaneous transcriptomic and spatial information on biopsy samples, in some cases down to a subcellular level. The range of spatial transcriptomic technologies and their technical principles have been comprehensively reviewed elsewhere (127). Integrating transcriptomic, proteomic, and genomic data with ST maximizes insight into the spatial heterogeneity of the LBCL TME and might reveal key signaling pathways and cell–cell interactions within defined cellular niches. The incorporation of complementary spatial information into existing single-cell technologies enables more in-depth functional characterization of cell subtypes, spatial organization patterns, key cellular interactions, and signaling pathways within the lymphoma TME. Harnessing knowledge gained from identifying and studying these functional multicellular neighborhoods may ultimately facilitate the identification of targetable inhibitory or suppressive mechanisms associated with immunotherapy resistance in future. No studies to date have reported ST analyses of the LBCL TME in CAR-T treated patients; however, some studies have used ST to describe aspects of the TME in a limited number of lymphoma subtypes.

In LBCL, ST has shown differences in T-cell transcriptional programs including chemo-attractant and chemo-inhibitory signals varying by their location within distinct cellular niches in LBCL biopsies. T-cells within niches enriched with other T-cells compared to either myeloid or tumor cells reflected a more naïve/memory phenotype with fewer exhaustion markers. T-cell exhaustion and dysfunction signatures varied by DLBCL subtype across LBCL, EBV-positive DLBCL, and DLBCLs of immune-privileged sites (128). Owing to their inherent structural and adherent properties, fibroblast and macrophage populations are difficult to isolate from tissue, leading to their frequent underrepresentation in scRNA-seq experiments. Dai et al. utilized ST in combination with bulk RNAseq to overcome this limitation and identified fibroblast and macrophage populations associated with prognosis following R-CHOP CIT, with the best OS seen in patients demonstrating higher levels of *FN1+* macrophages and *LYZ+* fibroblasts (129). In a multimodal analysis incorporating ST of 10 DLBCL samples prior to first-line CIT, a TAM cluster with increased glycolytic activity was identified. Compared with non-relapsed patients, those who relapsed exhibited higher levels of suppressive, interferon-primed TAMs (PD-L1^+^, PD-L2^+^, CXCL10^+^), increased PD-L1 expression, and enrichment of glycolysis-related gene expression, whereas non-relapsed patients demonstrated greater infiltration of activated CD8^+^ T cells (130). In a separate small study incorporating ST of 10 DLBCL patients, analysis of the TME validated findings from larger transcriptomic datasets, demonstrating higher *FAS* expression in non-relapsed compared with relapsed patients, particularly within Treg and Tfh cell populations (131).

Spatially resolved transcriptomic analysis using digital spatial profiling has further informed our understanding of tissue architecture in LBCL. Digital spatial profiling of CD68^+^ macrophage subsets in *de novo*, pre-treatment DLBCL biopsies, compared with reactive lymphoid tissue, identified macrophage signatures associated with clinical outcomes (132). Distinct macrophage signatures exhibited specific spatial localization within lymph nodes in DLBCL biopsies compared with reactive lymphoid tissue. The DLBCL-associated macrophage signature was characterized by upregulation of *CD163* and complement pattern-recognition components, with enrichment of interferon-γ and inflammatory response pathways. DLBCL cases enriched for either the DLBCL or dark zone macrophage signatures were associated with shorter OS. In another study of eight DLBCL cases, digital spatial profiling was used to compare transcriptional profiles of immune and stromal genes within regions of interest comprising stromal meshworks enriched for the mesenchymal markers smooth muscle actin or nerve growth factor receptor (133). These two stromal regions demonstrated differential expression of genes related to immune cell subsets (MDSCs, M2 macrophages), immune checkpoints, ECM and mesenchymal markers, highlighting the heterogeneity of stromal TME elements and their potential impact on tumor development.

Xia et al. utilized ST to examine immune infiltration patterns in primary CNS lymphoma (PCNSL) and their associations with cell communications and temporal TME changes during lymphoma evolution (134). Intercellular signaling patterns differed by TME subtype. For example, the hot tumor TME subtype, characterized by abundant intratumoral T-cell infiltration, demonstrated activation of the CXCR4–CXCL12 signaling axis, whereas the cold subtype, defined by sparse T-cell presence within the tumor and surrounding invasive margin, showed activation of CD99 signaling. Furthermore, expression of *CD274* (PD-L1) varied across TME subtypes and during tumor remodeling along TME trajectories and also displayed spatial heterogeneity. Distinct B-cell clusters with immunosuppressive features that colocalize with exhausted T cells have also been described in PCNSL (135). In follicular lymphoma, ST has enabled more precise characterization of immune cell subtypes, revealing distinct spatial and functional patterns of T-cell subsets in the TME (136). These insights have enhanced our understanding of disease biology and may provide valuable complementary prognostic information.

Current limitations of ST include the high associated costs and need for complex, specialized bioinformatic analysis of the substantial datasets generated. Transcript resolution varies across platforms, with some ST technologies capturing the whole transcriptome, whereas probe-based approaches profile limited, pre-defined gene sets. In addition, transcript capture efficiency differs between platforms and can affect detection of lowly expressed genes. Multimodal analyses of the LBCL TME to date have been limited by small cohorts undergoing ST, although this is likely to change going forward with the increasing availability of ST platforms. These technologies provide deeper insight into the spatial heterogeneity of the LBCL TME and add valuable information on the functional states of cell populations within their native tissue context. Rare and otherwise difficult-to-characterize cell subtypes can be interrogated using these approaches. Understanding the core mechanisms driving LBCL evolution and treatment resistance will, in turn, inform the development of novel targeted therapies. Analyses employing ST to investigate associations between the LBCL TME and CAR-T outcomes are awaited.

## 7 Discussion

In recent years, there has been an expansion in our understanding of how microenvironmental features in LBCL influence response to CD19 CAR-T therapy, yet substantial gaps in knowledge remain. PMBCL and tFL appear to be associated with more favorable outcomes following CD19 CAR-T therapy than other LBCL histologies, whereas molecular COO classification, genetic subtype and CD19 tumor expression levels have not demonstrated consistent associations with clinical response. Transcriptional programs reflecting a robust immune activation response following CAR-T infusion correlate with favorable outcomes, while enrichment of M2 macrophages and the close proximity of suppressive cell types to tumor cells or T-cells are associated with resistance. Interpretation across studies remains challenging due to small cohort sizes and significant methodological heterogeneity, underscoring the need for larger, standardized multimodal analyses.

The densely infiltrated lymph node architecture characteristic of LBCL poses significant challenges for spatial profiling, particularly for modalities such as multiplex immunofluorescence, where signal spillover and the constraints of current segmentation algorithms can impede accurate cellular resolution. In addition, the LBCL tumor microenvironment is composed of a diffuse admixture of malignant B-cells interspersed with diverse immune and stromal populations, rather than being organized into discrete tumor, stromal, vascular, or tertiary lymphoid regions commonly observed in other malignancies. In this context, analytical frameworks that define and interrogate functional cellular neighborhoods offer a powerful means of uncovering recurrent spatial patterns with potential clinical relevance. Continued advances in deep-learning computational methods are expected to streamline analysis of these highly complex, high-dimensional datasets and help overcome important limitations in existing tools. Emerging spatial omics technologies, including spatial transcriptomics, will further enhance our capacity to resolve the dynamic cellular ecosystems that drive tumor evolution and treatment resistance, complementing more established platforms.

Based on current insights into LBCL biology, the development of new CAR-T products and combination regimens that target immunosuppressive components of the TME is already progressing, with several candidates demonstrating early promise in the RR setting in clinical trials. A deeper understanding of the structural and functional organization of LBCL tissue architecture will be critical to guiding the rational design of these enhanced CAR-T therapies, ultimately enabling more effective strategies to overcome microenvironment-driven resistance and improve clinical outcomes.

### 7.1 Reprogramming the LBCL TME: clinical trials of CAR-Ts with novel approaches to overcome the immunosuppressive LBCL TME

Modifications to CD19 CAR-T therapy have been developed to counteract inhibitory TME mechanisms implicated in treatment resistance. Of the previously discussed inhibitory T-cell checkpoints (PD-1, TIGIT, LAG-3, TIM-3, CTLA-4, and BTLA), the PD-1/PD-L1 axis is the most well characterized in LBCL; and most studies modifying checkpoint inhibition in combination with CAR-Ts have investigated inhibition of the PD-1/PD-L1 axis (**Table 3**). Although PD-1/PD-L1 inhibition alone has shown limited success in DLBCL treatment previously, it was hypothesized that the response to CAR-Ts might be enhanced by reduction of CAR-T suppression caused by upregulated PD-L1 expression within the TME and the concomitant PD-1 expression of T-cells (155, 156). Methods of PD-1/PD-L1 inhibition include extrinsic suppression by administration of a PD-1 or PD-L1 immune checkpoint inhibitor (ICI) around the time of CAR-T infusion or by engineering CAR-Ts to resist PD-1/PD-L1 inhibition.

**Table 3:** Clinical studies investigating methods to overcome the immunosuppressive TME in CD19 CAR-T therapy.

Reference	Target axis and mechanism	Patient population	LD	Response rates	AE rates	Comments
Liu et al Clinical Cancer Research 2021 (137)	CD19 CAR-T with PD-1/CD28 switch receptor CAR-T dose 0.5–4 x10^6/kg	17 pts R/R lymphoma 13 DLBCL 2 MCL 2 tFL	Cyclo 500mg/m2/d + Flu 30mg/m2/d D-5 to D-3	3m ORR: 58.8% CRR: 41.2% At median 15m FU, median OS NR	CRS: G1 47.06% G2 41.18% No G3–4 CRS No NT observed	PD-L1 H score >10 predictive of OS
Hu et al eClinicalMedicine 2023 (138)	CD19 CAR-T with non-viral CRISPR/Cas9 integration into PD-1 CAR-T dose escalation: 2/4/6 x10^6/kg 0.56-0.8x10^6/kg (non-standard)	21 pts R/R B-NHL 17 DLBCL 2 MCL 1 B-LBL 1 FL	Cyclo 500mg/m2/d D-3 to D-2 Flu 30mg/m2/d D-4 to D-2	ORR: 100% CRR: 81% Median PFS: 19.5m Median OS: NR 12m OS: 76%	G1–2 CRS: 67% G1–2 ICANS: 19% No G3–4 CRS/ICANS	Pre-treatment PD-L1 expression detected in 11/17 pts (65%) with available data 4 pts >50% expression – 3/4 pts achieved and maintained CR
Kim et al Haematological Oncology (abstract) 2023 (139)	CD19 CAR-T with knockout of PD-1 and TIGIT expression CAR-T dose 2x10^6/kg	41 pts RR LBCL	LD not described	ORR: 85.3% D28 CRR: 73.5% 3m CRR: 71.4%	G3 CRS 14.6% G3 ICANS 4.9% No G4 CRS/ICANS	Responder group had higher levels of CCR7+ memory phenotype and higher PD-1 knockout rate
Liu et al Translational Oncology 2021 (140)	CD19 CAR-T with dominant negative mutated PD-1 sequence expressing inactive PD-1 CAR-T dose >1x10^6/kg	9 pts RR B-NHL 4 DLBCL 2 tFL 3 FL	Cyclo 500mg/m2/d + Flu 30mg/m2/d D-5 to D-3	D30 ORR: 77.8% D30 CRR: 22.2% Overall CRR: 55.6% In DLBCL pts – 1 CR (25%) and 2 PR (50%)	All grade CRS 100% G3 CRS 11.1% All grade NT 11.1% No G3/4 ICANS	PD-L1 detected (>20% by IHC) in 3 pts – 1/3 pts achieved CR
Hu et al Journal of Clinical Oncology (abstract) 2024 (141)	Allogeneic CD19 CAR-T with knockout of TRAC and PDCD1 genes CAR-T dose escalation: 40/80/120x10^6	16 pts RR B-NHL 10 LBCL 3 MCL 2 FL POD24 1 MZL	Cyclo 60mg/kg/d for 2d Flu 25mg/m2/d for 5d	All pts vs LBCL cohort: ORR: 94% vs 90% CRR: 69% vs 70% CRR at >6m: 44% vs 50%	All grade CRS 44% No G3/4 CRS All grade ICANS 25% ICANS G≥3 13% No GVHD No G3/4 CRS	
Wang et al International Immunopharmacology 2024 (142)	CD19/22 cocktail CAR-T +/- ASCT PD-1 inhibitor and chidamide maintenance CAR19 dose 0.7-12.6x10^6/kg CAR20 dose 0.8-10x10^6/kg	52 pts RR aggressive LBCL 95 DLBCL, NOS 9 HGBL	Cyclo 300mg/m2/d + Flu 25mg/m2/d D-4 to D-2	Treatment vs control group (PD-1 and chidamide not received): ORR: 75% vs 77% CRR: 46% vs 54% 5Y OS: 89% vs 59% 5Y PFS: 77% vs 46%	CRS and ICANS rates not reported	Baseline tumor PD-L1 expression not reported
Hirayama et al Blood Advances 2024 (143)	CD19 CAR-T + PD-L1 inhibitor (durvalumab) starting either after CAR-T or 1d prior to infusion CAR-T dose 2x10^6/kg	29 pts RR LBCL 13 DLBCL, NOS 8 tDLBCL 6 HGBL 1 PMBCL 1 RT	Cyclo 300mg/m2/d + Flu 25/m2/d for 3d, completed 2-5d pre CAR-T infusion	Best ORR: 38% Best CRR: 35% 3m ORR: 35% 3m CRR: 27% At median 44.5m FU, G1 vs G2: 2Y PFS: 22% and 23.5% 2Y OS: 55.6% and 52.9%	Group 1 vs Group: Any grade CRS: 45% vs 39% G≥3 CRS: 9% vs 6% Any grade NT: 27% vs 11% G≥3 NT: 9% vs 6%	Better DOR in CAR-T + PD-L1 than CAR-T alone Late conversions to CR with maintenance PD-L1 ICI
Jaeger et al Blood Advances 2023 (144)	Tisa-cel + PD-1 inhibitor (pembrolizumab) PD-1 commencing D + 15, D + 8 or D-1 CAR-T dose not described	12 pts RR DLBCL	LD not described	CRR: 33.3% PR: 17% PD: 50%	All grade CRS 58.3% G≥3 CRS 8.3% All grade ICANS 8.3% G≥3 ICANS 0%	Highest response rate at D-1 PD-1 administration– ORR 75%, 3 sustained CR
Xue et al Blood (abstract) 2023 (145)	CD19 or CD20 CAR-T + PD-1 inhibition (sintilimab or tislelizumab) after infusion CAR-T dose 1-3x10^6/kg	22 pts RR DLBCL with TP53 alterations (mutations/deletions)	LD not described	CAR only group vs CAR+ICI group: CRR 7.7% vs 66.7% Median PFS 1.7m vs NR Median OS 10.9m vs NR	G3–4 CRS 4.5% No ICANS	
Mu et al Hematol Oncol 2023 (146)	CD19 CAR-T + PD-1 inhibitor (sintilimab) starting D-1 pre-infusion then maintenance if CR/PR achieved CAR-T dose 2x10^6/kg	44 pts RR DLBCL with high tumor burden (1 evaluable lesion >7.5cm)	Cyclo 400mg/m2/d + Flu 30mg/m2/d D-4 to D-2	CAR only group vs CAR+ICI group: ORR: 61% vs 65% CRR: 39% vs 42% 3m PFS: 33% vs 81% 12m PFS: 27% vs 47% 3m OS: 61% vs 92% 12m OS: 39% vs 65%	CAR only group vs CAR+ICI group: All grade CRS: 72.23% vs 84.61% G≥3 CRS: 5.56% vs 15.38% All grade ICANS: 11.11% vs 11.54% G≥3 ICANS: 0% vs 0%	No significant difference in CAR-T response by pre-treatment PD-1 expression on T-cells
Jacobson et al Cancer Research (abstract) 2020 (147)	Axi-cel + PD-L1 inhibitor (atezolizumab) after infusion starting either D + 1, D + 14 or D + 21 CAR-T dose 2x10^6/kg	28 pts Refractory DLBCL	Cyclo 500mg/m2/d + Flu 30mg/m2/d for 3d	Best ORR: 75% CRR: 46% Median DOR/PFS/OS NR at 10.2 FU	G≥3 CRS 4% G≥3 NT 29% G5 MODS 3.6%	Similar efficacy outcomes of CAR-T + ICI to axi-cel alone as reported in ZUMA-1 trial
Cao et al Frontiers in Oncology 2019 (148)	CD19 CAR-T + PD-1 inhibitor (nivolumab) single dose on D + 3 CAR-T dose 8x10^6/kg (range 5-11)	11 pts RR B-NHL 10 DLBCL 1 Burkitt’s	Cyclo 500mg/m2/d + Flu 30mg/m2/d for 3d	ORR: 81% CRR: 45% Median PFS: 6m	G1–2 CRS 81.8% No G>3 CRS or NT NT – tremors 1 pt (grade not described)	No relationship between PD-L1 tumor expression by IHC and early relapse/no response
Siddiqi et al Haem Onc (abstract) 2019 (149)	Liso-cel + PD-L1 inhibitor (durvalumab) from D + 29 CAR-T dose escalation: 50 or 100 x10^6	18 pts 11 pts received PD-L1 RR B-NHL 10 DLBCL 1 G3B FL	Cyclo + Flu for 3d (dose not described)	Best ORR: 91% CRR: 64%	No CRS after PD-L1 inhibitor infusion G1–2 NT rates not specified No G≥3 NT	
Osborne et al Journal of Clinical Oncology (abstract) 2020 (150)	CD19/22 bicistronic CAR-T (AUTO3) + PD-1 inhibitor (pembrolizumab) PD-1 given either starting on D14 or single dose on D-1 CAR-T dose escalation: 50/150/450 x10^6	19 pts RR DLBCL, NOS or tDLBCL (pt numbers not specified)	Cyclo + Flu (dose/timing not described)	In pts receiving DLT2/3: ORR: 64% CRR: 55%	No severe CRS Severe NT 5% Grading not specified	
Ahmed et al Blood (abstract) 2024 (151)	CD19 CAR-T + IL-15 polymer-conjugated agonist (NKTR-255) from D + 14 q21d up to 5m CAR-T dose not reported	15 pts 11 NKTR-255 group 4 placebo RR LBCL	LD not reported	CAR-T + IL-15 vs CAR-T alone: 6m CRR: 73% vs 50% Conversion to CR from SD/PR at 6m: 25% vs 0%	CRS and NT rates not reported	Re-expansion of CD8+ CAR-Ts occurred in 73% of NKTR-255 group vs 0% in placebo group
Svoboda et al New England Journal of Medicine 2025 (152)	CD19 armored CAR-T with constitutive IL-18 secretion with humanized scFv CAR-T dose 3x10^6 – 3x10^8 3 day manufacturing process	21 pts (5 retreated) RR B-NHL 8 DLBCL 2 tFL 1 HGBL 1 THRBCL 6 FL 3 MCL	Benda 90mg/m2/d for 2d OR Cyclo 250mg/m2/d + Flu 25mg/m2/d for 3d	3m ORR: 81% CRR: 52% LBCL cohort ORR: 67% Median DOR: 9.6m (at 17.5m) Median duration of PFS: 8.7m	All grade CRS 62% CRS G1-2 47% CRS G3 14% ICANS G1/2 14% NO G≥3 ICANS No IEC-HS	Response rates lower in pts previously treated with 41BB product vs CD28
Lei et al Cell discovery 2024 (153)	CD19 armored CAR expressing IL-7 and CCL-19 upon CD19 engagement CAR-T dose escalation: 0.5/1/2/4x10^6/kg	39 pts RR LBCL 33 DLBCL 1 PMBCL 2 tFL 3 MCL	Cyclo 500mg/m2/d + Flu 30mg/m2/d D-5 to D-3	ORR 79.5% Best CRR 61.5% Median PFS 13m Median OS NR For CR pts: Median DOR NR Est 12m OS 95.8% Est 24m OS 74.3%	All grade CRS 74.4% CRS G3 12.8% All grade ICANS 10.3% G≥3 10.3%	Pts with higher peak IL-7 and CCL-19 had better OS and PFS
Palomba et al Blood (abstract) 2024 (154)	CD19 CAR-T with mutated IL-2 receptor + mutant CAR-T binding IL-2 administration started pre-infusion CAR-T dose 30x10^6 or 90x10^6	6 pts 1 DLBCL 3 CLL 2 MZL RR CD19+ hematological malignancies	Cyclo 300mg/m2/d + Flu 30mg/m2/d D-5 to D-3	DLBCL pt in CR for 90+ days	CRS G1-2 33% ICANS G1-2 17% No G≥3 CRS or ICANS	

B-LBL, B-lymphoblastic lymphoma; B-NHL, B-cell Non-Hodgkin Lymphoma; CLL, Chronic Lymphocytic Leukemia; CR, Complete Response; CRR, Complete Response Rate; CRISPR, Clustered Regularly Interspaced Short Palindromic Repeats; CRS, Cytokine Release Syndrome; Cyclo, Cyclophosphamide; d, Day; DLBCL, Diffuse Large B-cell Lymphoma; DLBCL NOS, Diffuse Large B-cell Lymphoma, Not Otherwise Specified; DOR, Duration of Response; FL, Follicular Lymphoma; FL POD24, Follicular Lymphoma with Progression of Disease within 24 months; Flu, Fludarabine; FU, Follow-up; G, Grade; GVHD, Graft-versus-Host Disease; HGBL, High-Grade B-cell Lymphoma; ICANS, Immune Effector Cell-Associated Neurotoxicity Syndrome; ICI, Immune Checkpoint Inhibitor; IEC-HS, Immune Effector Cell-Associated Hemophagocytic Lymphohistiocytosis-like Syndrome; LBCL, Large B-cell Lymphoma; LD, Lymphodepletion; MCL, Mantle Cell Lymphoma; MODS, Multiple Organ Dysfunction Syndrome; m, Month; MZL, Marginal Zone Lymphoma; NR, No Response; NT, Neurotoxicity; ORR, Overall Response Rate; OS, Overall Survival; PD, Progressive Disease; PFS, Progression-Free Survival; PR, Partial Response; PMBCL, Primary Mediastinal B-cell Lymphoma; RT, Richter Transformation; scFv (Single-chain Variable Fragment); SD, Stable Disease; tDLBCL, Transformed Diffuse Large B-cell Lymphoma; tFL, Transformed Follicular Lymphoma; THRLBCL, T-cell/Histiocyte-rich Large B-cell Lymphoma.

Administration of PD-1/PD-L1 ICIs around the time of CD19 CAR-T infusion has demonstrated varying outcomes across published studies. There is no consistent evidence that adjuvant PD-1/PD-L1 ICI administration improves clinical responses above that of CD19 CAR-T therapy alone, although many of the published studies are only available in abstract form or include small patient numbers (142–150). Timing of administration and ICI choice may impact clinical outcomes (143, 144). ICI administration may play a role in achieving late term conversion to CR or contribute to maintaining a response in those who achieve CR (143). The addition of PD-1/PD-L1 inhibition does not appear to significantly increase rates of CAR-T specific AEs such as CRS or ICANS, although ICIs are associated with their own unique AE profile. Extrinsic PD-1/PD-L1 ICI administration may augment CAR-T expansion dynamics by causing secondary CAR-T re-expansion, although this finding has not been consistently observed across the aforementioned studies (143, 144, 146, 147, 149). Whether pre-treatment PD-L1 expression levels correlate with response to extrinsic or intrinsic PD-1/PD-L1 inhibition remains unclear with different findings reported across available trials (137, 148, 157, 158). Longer term data with larger patient cohorts are required to confirm early findings suggesting that intrinsic CAR-T PD-1 inhibition may enhance clinical responses without increasing toxicity (137, 139–141, 158). Small studies and case reports examining treatment with PD-1 or PD-L1 inhibition following LBCL relapse after CAR-T administration have not demonstrated significant benefit (156, 157, 159, 160).CD20 CAR-Ts with OX40 co-expression also show promising proliferation kinetics and effector function in DLBCL patients without significant toxicity (161).

Inhibitory cytokines within the LBCL TME can promote tumor growth via induction of immunosuppressive cell subtypes, such as by triggering M2 macrophage polarization, and reduce anti-tumor immune responses by dampening the effector function of cell subtypes in the TME. The development of cytokine-engineered CAR-Ts to overcome complications of systemic cytokine administration and directly target the local immune milieu within the TME shows promise in enhancing CAR-T efficacy for LBCL. Fourth-generation CAR-Ts engineered to secrete IL-18, IL-7 or CCL-19 have demonstrated encouraging preliminary results in early-phase clinical trials (152, 153). The co-administration of exogenous pegylated IL-15 or mutated IL-2 with CAR-Ts has also shown promising outcomes (151, 154). Generation of lymphoma organoid models may allow further *in vitro* investigation into causes of resistance to immunotherapies in future and permit preclinical testing of novel treatment combinations or augmentations to CAR-T products (162).

### 7.2 Reprogramming the LBCL TME: preclinical approaches to modulating CAR-Ts to overcome the TME

Preclinical studies have investigated inhibition of checkpoint proteins using multiple strategies to enhance CAR-T efficacy by targeting suppressive elements the TME (163–167). Agarwal et al. demonstrated that CRISPR-Cas9 disruption of CTLA-4 expression in CD19 CAR-Ts improved anti-tumor activity compared with unmodified CD19 CAR-Ts or combined PD-1/CTLA-4 or PD-1-only knockouts in xenograft models of B-cell acute lymphoblastic leukemia and myeloma (163). In contrast, CRISPR-Cas9 knockout of LAG-3 did not improve efficacy beyond standard CD19 CAR-Ts in a separate murine lymphoma xenograft model (164).

Dual checkpoint modulation has also been explored, with combined PD-1 and TIGIT downregulation resulting in superior anti-tumor responses compared with PD-1 knockdown paired with TIM-3, LAG-3 or CTLA-4 inhibition (168). Alternative approaches including CAR-T secretion of anti-PD-1 molecules improved CAR proliferation and persistence *in vitro* (169). TIM-3/CD28 switch-receptor incorporation into CD19 CAR-Ts enhanced CAR-T function *in vitro* and improved persistence and anti-tumor efficacy in murine models without significant CRS or ICANS (165, 166).

Preclinical studies have also explored engineered CAR-Ts that secrete cytokines within the TME, either constitutively or in response to triggers such as antigen engagement or local cues such as switch-receptor activation and hypoxia. IL-15 is a homeostatic cytokine that plays a critical role in T-cell survival and in the maintenance of CD8+ memory T-cells and NK cells. Several studies have leveraged IL-15 to enhance CAR-T expansion, persistence and anti-tumor activity in the TME.

Conditional secretion of IL-15 by CD19 CAR-Ts following antigen engagement improved survival, expansion and anti-tumor activity compared to conventional CD19 CAR-Ts, while systemic co-administration of polymer-conjugated IL-15 similarly enhanced activity in murine models (170, 171). However, sustained autocrine IL-15 signaling achieved through expression of an IL15­IL15Rα fusion protein on CD19 CAR-Ts was associated with significant systemic toxicity in immunocompetent BALB/c mice (172). Together these findings indicate that while IL-15 potently augments CAR-T function, the optimal strategy for exploiting its benefits likely requires tight spatial and temporal control to avoid unacceptable toxicity.

IL-21 is a pleiotropic cytokine that enhances T-cell proliferation and effector function while preserving an early memory-like T-cell phenotype and limiting terminal differentiation. Stach et al. found that CD19 CAR-Ts engineered to secrete IL-21 upon activation showed enhanced anti-tumor effects and tumor infiltration compared to CD19 CAR-Ts *in vivo* (173). An early study by Markley et al. compared constitutive expression of IL-2, IL-7, IL-15 or IL-21 by CD19 CAR-Ts and demonstrated distinct effects on T-cell differentiation, persistence and efficacy, with IL-7 and IL-21 conferring superior *in vivo* lymphoma eradication compared with IL-2 and IL-15 (174).

IL-12 is a pro-inflammatory cytokine that induces IFN-γ production by T-cells and promotes Th1 polarization and may show promise in augmenting CAR-T anti-tumor responses (175). CD19 CAR-Ts secreting IL-36γ have also demonstrated enhanced tumor eradication and persistence in xenograft models, accompanied by activation of an endogenous antitumor response (176).

Harnessing suppressive signals within the TME and redirecting them toward anti-tumor immune responses has been explored. TGF-β signaling within the TME suppresses T-cell activation and proliferation, inhibits CD4 and CD8 effector function and promotes Treg differentiation, whereas IL-7 plays a central role in T-cell development, expansion, differentiation and survival. CD19 CAR-Ts engineered to express a TGF-β/IL-7 switch-receptor to convert suppressive TME signals into pro-inflammatory responses improved survival *in vivo* (177).

Metabolic features of an adverse TME, including hypoxia, nutrient deprivation and an acidic microenvironment, further constrain anti-tumor immune responses. While LBCL tumor cells adapt to tolerate these stressors, immune effector cells are functionally impaired. Zhou et al. developed a CD19 CAR-T construct that conditionally secretes IL-12 in hypoxic environments, resulting in enhanced anti-tumor efficacy without significant toxicity in preclinical models (178).

Metabolites secreted into the TME by local cells can also inhibit CAR-T function; however there are comparatively fewer studies examining strategies to overcome this form of immunosuppression in LBCL than in solid organ malignancies. Indoleamine 2,3-dioxygenase (IDO) is an intracellular enzyme involved in the conversion of tryptophan into the inhibitory metabolites kynurenine and 3-HAA that suppresses CD19 CAR-T efficacy *in vitro* and represents a potential target for future CAR-T approaches aimed at locally mitigating IDO-mediated suppression within the TME (179).

Arginine is an amino acid metabolized by arginase-1 and -2 (ARG1, ARG2), enzymes produced by suppressive myeloid populations within the TME, including MDSCs and TAMs, resulting in impaired T-cell responses. While preclinical studies in solid tumors have explored targeting this pathway using novel CAR-T engineering strategies, analogous investigations in LBCL have yet to be reported (180).

Adenosine is another immunosuppressive metabolite within the TME, generated by extracellular ATP catabolism via CD39 and CD73, and inhibits multiple anti-tumor immune populations including T-cells, NK cells, macrophages and DCs (181). Although the adenosine pathway is better characterized in solid organ malignancies, it represents a promising avenue for future investigation in LBCL.

While cancer-associated fibroblasts are associated with poorer outcomes in LBCL, direct fibroblast-targeting strategies have not yet been evaluated in the context of CD19 CAR-T in LBCL. A BCMA-directed CAR-T designed to concomitantly target fibroblast antigens, including fibroblast activation protein (FAP) or signaling lymphocyte activation molecule family-7 (SLAMF7), improved survival compared to conventional BCMA CAR-T in a myeloma xenograft model (182). Emerging evidence also suggests that the gut microbiome may influence CAR-T outcomes and further studies may elucidate whether specific microbial compositions or metabolites could serve as predictive biomarkers in LBCL (183).

Collectively, these preclinical studies demonstrate that CAR-Ts can be engineered to resist or reprogram suppressive mechanisms within the LBCL TME, including through checkpoint modulation, switch-receptor signaling and localized delivery of pro-inflammatory cytokines. Given the pleiotropic effects of many cytokines, particularly with systemic exposure, careful mechanistic understanding and cautious toxicity assessment remain essential. Numerous *ex vivo* CAR-T manufacturing strategies have been described elsewhere and are beyond the scope of this review. Finally, the limited availability of preclinical models that accurately recapitulate the complexity of the LBCL TME remains a major challenge for translational investigation in this area.

### 7.3 Future directions

The development of CD19 CAR-T therapy represents a substantial advance in the treatment of R/R LBCL, providing durable remissions in a subset of patients with R/R disease. Analysis of the factors leading to treatment failure is paramount to improving patient outcomes. An immunosuppressive TME presents a barrier to CAR-T efficacy by impairing CAR-T infiltration and promoting CAR-T dysfunction and poor survival, in addition to detrimental effects on endogenous T-cells. With the increasing availability of high dimensional techniques to study the complex lymphoma TME, multimodal approaches will provide complementary information to inform our understanding of facets of the LBCL TME that drive immune evasion and treatment resistance. These studies will provide insights required to design new approaches to optimize CAR-T immunotherapies for LBCL.

## References

[1] Flowers CR Sinha R Vose JM . Improving outcomes for patients with diffuse large B-cell lymphoma. Ca: A Cancer J For Clin. (2010) 60:393–408. doi: 10.3322/caac.20087 21030533

[2] Alaggio R Amador C Anagnostopoulos I Attygalle AD Araujo I Berti E . The 5th edition of the World Health Organization classification of haematolymphoid tumours: Lymphoid neoplasms. Leukemia. (2022) 36:1720–48. doi: 10.1038/s41375-022-01620-2 35732829 PMC9214472

[3] Crump M Neelapu SS Farooq U Van Den Neste E Kuruvilla J Westin J . Outcomes in refractory diffuse large B-cell lymphoma: Results from the international SCHOLAR-1 study. Blood. (2017) 130:1800–8. doi: 10.1182/blood-2017-03-769620 28774879 PMC5649550

[4] Sehn LH Salles G . Diffuse large B-cell lymphoma. N Engl J Med. (2021) 384:842–58. doi: 10.1016/j.hoc.2016.07.010 33657296 PMC8377611

[5] Neelapu SS Locke FL Bartlett NL Lekakis LJ Miklos DB Jacobson CA . Axicabtagene ciloleucel CAR T-cell therapy in refractory large B-cell lymphoma. N Engl J Med. (2017) 377:2531–44. doi: 10.1056/nejmoa1707447 29226797 PMC5882485

[6] Schuster SJ Bishop MR Tam CS Waller EK Borchmann P McGuirk JP . Tisagenlecleucel in adult relapsed or refractory diffuse large B-cell lymphoma. N Engl J Med. (2018) 380:45–56. doi: 10.1056/nejmoa1804980 30501490

[7] Locke FL Miklos DB Jacobson CA Perales M-A Kersten M-J Oluwole OO . Axicabtagene ciloleucel as second-line therapy for large B-cell lymphoma. N Engl J Med. (2021) 386:640–54. doi: 10.1056/nejmoa2116133 34891224

[8] Kamdar M Solomon SR Arnason J Johnston PB Glass B Bachanova V . Lisocabtagene maraleucel versus standard of care with salvage chemotherapy followed by autologous stem cell transplantation as second-line treatment in patients with relapsed or refractory large B-cell lymphoma (TRANSFORM): Results from an interim analysis of an open-label, randomised, phase 3 trial. Lancet. (2022) 399:2294–308. doi: 10.1016/s0140-6736(22)00662-6 35717989

[9] Maude SL Laetsch TW Buechner J Rives S Boyer M Bittencourt H . Tisagenlecleucel in children and young adults with B-cell lymphoblastic leukemia. N Engl J Med. (2018) 378:439–48. doi: 10.1056/nejmoa1709866 29385370 PMC5996391

[10] Shah BD Bishop MR Oluwole OO Logan AC Baer MR Donnellan WB . KTE-X19 anti-CD19 CAR T-cell therapy in adult relapsed/refractory acute lymphoblastic leukemia: ZUMA-3 phase 1 results. Blood. (2021) 138:11–22. doi: 10.1182/blood.2020009098 33827116 PMC9999039

[11] Rodriguez-Otero P Ailawadhi S Arnulf B Patel K Cavo M Nooka AK . Ide-cel or standard regimens in relapsed and refractory multiple myeloma. N Engl J Med. (2023) 388:1002–14. doi: 10.1056/nejmoa2213614 36762851

[12] San-Miguel J Dhakal B Yong K Spencer A Anguille S Mateos M-V . Cilta-cel or standard care in lenalidomide-refractory multiple myeloma. N Engl J Med. (2023) 389:335–47. doi: 10.1056/nejmoa2303379 37272512

[13] Abramson JS Palomba ML Gordon LI Lunning MA Wang M Arnason J . Lisocabtagene maraleucel for patients with relapsed or refractory large B-cell lymphomas (TRANSCEND NHL 001): A multicentre seamless design study. Lancet. (2020) 396:839–52. doi: 10.1016/s0140-6736(20)31366-0 32888407

[14] Amini L Silbert SK Maude SL Nastoupil LJ Ramos CA Brentjens RJ . Preparing for CAR T cell therapy: Patient selection, bridging therapies and lymphodepletion. Nat Rev Clin Oncol. (2022) 19:342–55. doi: 10.1038/s41571-022-00607-3 35318469

[15] Turtle CJ Hanafi LA Berger C Hudecek M Pender B Robinson E . Immunotherapy of non-Hodgkin's lymphoma with a defined ratio of CD8+ and CD4+ CD19-specific chimeric antigen receptor-modified T cells. Sci Transl Med. (2016) 8:355ra116. doi: 10.1126/scitranslmed.aaf8621 27605551 PMC5045301

[16] Sterner RC Sterner RM . CAR-T cell therapy: Current limitations and potential strategies. Blood Cancer J. (2021) 11:69. doi: 10.1038/s41408-021-00459-7 33824268 PMC8024391

[17] Agency for Clinical Innovation NG . Eligibility and referral: Indications for CAR-T treatment. Available online at: https://aci.health.nsw.gov.au/projects/immune-effector-cell-service/eligibility-and-referral (Accessed October 28, 2024).

[18] Administration UFaD . Cellular and gene therapy products: BREYANZI (lisocabtagene maraleucel) (2024). Available online at: https://www.fda.gov/vaccines-blood-biologics/cellular-gene-therapy-products/breyanzi-lisocabtagene-maraleucel (Accessed October 28, 2024).

[19] Administration UFaD . Cellular and gene therapy products: KYMRIAH (tisagenlecleucel) (2024). Available online at: https://www.fda.gov/vaccines-blood-biologics/cellular-gene-therapy-products/kymriah (Accessed June 15, 2025).

[20] Caballero AC Escribà-Garcia L Alvarez-Fernández C Briones J . CAR T-cell therapy predictive response markers in diffuse large B-cell lymphoma and therapeutic options after CART19 failure. Front Immunol. (2022) 13:904497. doi: 10.3389/fimmu.2022.904497 35874685 PMC9299440

[21] Gong IY Tran D Saibil S Laister RC Kuruvilla J . Biomarkers of outcome in patients undergoing CD19 CAR-T therapy for large B cell lymphoma. HemaSphere. (2024) 8:e130. doi: 10.1002/hem3.130 39175824 PMC11339649

[22] Locke FL Rossi JM Neelapu SS Jacobson CA Miklos DB Ghobadi A . Tumor burden, inflammation, and product attributes determine outcomes of axicabtagene ciloleucel in large B-cell lymphoma. Blood Adv. (2020) 4:4898–911. doi: 10.1182/bloodadvances.2020002394 33035333 PMC7556133

[23] Deng Q Han G Puebla-Osorio N Ma MCJ Strati P Chasen B . Characteristics of anti-CD19 CAR T cell infusion products associated with efficacy and toxicity in patients with large B cell lymphomas. Nat Med. (2020) 26:1878–87. doi: 10.1038/s41591-020-1061-7 33020644 PMC8446909

[24] Scott DW Gascoyne RD . The tumour microenvironment in B cell lymphomas. Nat Rev Cancer. (2014) 14:517–34. doi: 10.1038/nrc3774 25008267

[25] Yang J Chen Y Jing Y Green MR Han L . Advancing CAR T cell therapy through the use of multidimensional omics data. Nat Rev Clin Oncol. (2023) 20:211–28. doi: 10.1038/s41571-023-00729-2 36721024 PMC11734589

[26] Sinkarevs S Strumfs B Volkova S Strumfa I . Tumour microenvironment: The general principles of pathogenesis and implications in diffuse large B cell lymphoma. Cells. (2024) 13:1057. doi: 10.3390/cells13121057 38920685 PMC11201569

[27] Zhao Y Shen M Wu L Yang H Yao Y Yang Q . Stromal cells in the tumor microenvironment: Accomplices of tumor progression? Cell Death Dis. (2023) 14:587. doi: 10.1038/s41419-023-06110-6 37666813 PMC10477351

[28] Galon J Bruni D . Approaches to treat immune hot, altered and cold tumours with combination immunotherapies. Nat Rev Drug Discov. (2019) 18:197–218. doi: 10.1038/s41573-018-0007-y 30610226

[29] Castro F Cardoso AP Gonçalves RM Serre K Oliveira MJ . Interferon-gamma at the crossroads of tumor immune surveillance or evasion. Front Immunol. (2018) 9. doi: 10.3389/fimmu.2018.00847 29780381 PMC5945880

[30] Kohli K Pillarisetty VG Kim TS . Key chemokines direct migration of immune cells in solid tumors. Cancer Gene Ther. (2022) 29:10–21. doi: 10.1038/s41417-021-00303-x 33603130 PMC8761573

[31] Ng WL Ansell SM Mondello P . Insights into the tumor microenvironment of B cell lymphoma. J Exp Clin Cancer Res. (2022) 41:362. doi: 10.1186/s13046-022-02579-9 36578079 PMC9798587

[32] He X Xu C . Immune checkpoint signaling and cancer immunotherapy. Cell Res. (2020) 30:660–9. doi: 10.1038/s41422-020-0343-4 32467592 PMC7395714

[33] Strizova Z Benesova I Bartolini R Novysedlak R Cecrdlova E Foley LK . M1/M2 macrophages and their overlaps - myth or reality? Clin Sci (Lond). (2023) 137:1067–93. doi: 10.1042/cs20220531 37530555 PMC10407193

[34] Yu R Zhu B Chen D . Type I interferon-mediated tumor immunity and its role in immunotherapy. Cell Mol Life Sci. (2022) 79:191. doi: 10.1007/s00018-022-04219-z 35292881 PMC8924142

[35] Xiong X Xie X Wang Z Zhang Y Wang L . Tumor-associated macrophages in lymphoma: From mechanisms to therapy. Int Immunopharmacol. (2022) 112:109235. doi: 10.1016/j.intimp.2022.109235 36215869

[36] Liu Y Zhou X Wang X . Targeting the tumor microenvironment in B-cell lymphoma: Challenges and opportunities. J Hematol Oncol. (2021) 14:125. doi: 10.1186/s13045-021-01134-x 34404434 PMC8369706

[37] DhatChinamoorthy K Colbert JD Rock KL . Cancer immune evasion through loss of MHC class I antigen presentation. Front Immunol. (2021) 12. doi: 10.3389/fimmu.2021.636568 33767702 PMC7986854

[38] Wu J Meng F Ran D Song Y Dang Y Lai F . The metabolism and immune environment in diffuse large B-cell lymphoma. Metabolites. (2023) 13:734. doi: 10.3390/metabo13060734 37367892 PMC10300995

[39] Pangarsa EA Rizky D Tandarto K Naibaho RM Kurniawan SP Istiadi H . The expression of hypoxia inducible factor-1 alpha in diffuse large B-cell lymphoma (DLBCL) patients: A cross-sectional study in Indonesia. Ann Med Surg. (2023) 85:4780–7. doi: 10.1097/ms9.0000000000001162 37811023 PMC10553143

[40] Bhalla K Jaber S Nahid MN Underwood K Beheshti A Landon A . Role of hypoxia in diffuse large B-cell lymphoma: Metabolic repression and selective translation of HK2 facilitates development of DLBCL. Sci Rep. (2018) 8:744. doi: 10.1038/s41598-018-19182-8 29335581 PMC5768748

[41] Huang J Zhang L Wan D Zhou L Zheng S Lin S . Extracellular matrix and its therapeutic potential for cancer treatment. Signal Transduction Targeted Ther. (2021) 6:153. doi: 10.1038/s41392-021-00544-0 33888679 PMC8062524

[42] Steen CB Luca BA Esfahani MS Azizi A Sworder BJ Nabet BY . The landscape of tumor cell states and ecosystems in diffuse large B cell lymphoma. Cancer Cell. (2021) 39:1422–1437.e10. doi: 10.1016/j.ccell.2021.08.011 34597589 PMC9205168

[43] Kotlov N Bagaev A Revuelta MV Phillip JM Cacciapuoti MT Antysheva Z . Clinical and biological subtypes of B-cell lymphoma revealed by microenvironmental signatures. Cancer Discov. (2021) 11:1468–89. doi: 10.1158/2159-8290.cd-20-0839 33541860 PMC8178179

[44] Li X Singhal K Deng Q Chihara D Russler-Germain D Harkins RA . Large B cell lymphoma microenvironment archetype profiles. Cancer Cell. (2025) 43:1347–1364.e13. doi: 10.1016/j.ccell.2025.06.002 40920660 PMC12417684

[45] Lenz G Wright G Dave SS Xiao W Powell J Zhao H . Stromal gene signatures in large-B-cell lymphomas. N Engl J Med. (2008) 359:2313–23. doi: 10.1056/nejmoa0802885 19038878 PMC9103713

[46] Autio M Leivonen S-K Brück O Karjalainen-Lindsberg M-L Pellinen T Leppä S . Clinical impact of immune cells and their spatial interactions in diffuse large B-cell lymphoma microenvironment. Clin Cancer Res. (2022) 28:781–92. doi: 10.1158/1078-0432.ccr-21-3140 34907083 PMC9377736

[47] Matias A Suvi-Katri L Oscar B Satu M Judit Mészáros J Marja-Liisa K-L . Immune cell constitution in the tumor microenvironment predicts the outcome in diffuse large B-cell lymphoma. Haematologica. (2020) 106:718–29. doi: 10.3324/haematol.2019.243626 32079690 PMC7927991

[48] Xu-Monette ZY Xiao M Au Q Padmanabhan R Xu B Hoe N . Immune profiling and quantitative analysis decipher the clinical role of immune-checkpoint expression in the tumor immune microenvironment of DLBCL. Cancer Immunol Res. (2019) 7:644–57. doi: 10.1158/2326-6066.cir-18-0439 30745366

[49] Nam SJ Go H Paik JH Kim TM Heo DS Kim CW . An increase of M2 macrophages predicts poor prognosis in patients with diffuse large B-cell lymphoma treated with rituximab, cyclophosphamide, doxorubicin, vincristine and prednisone. Leukemia Lymphoma. (2014) 55:2466–76. doi: 10.3109/10428194.2013.879713 24397616

[50] Wright KT Weirather JL Jiang S Kao KZ Sigal Y Giobbie-Hurder A . Diffuse large B-cell lymphomas have spatially defined, tumor immune microenvironments revealed by high-parameter imaging. Blood Adv. (2023) 7:4633–46. doi: 10.1182/bloodadvances.2023009813 37196647 PMC10448427

[51] Colombo AR Hav M Singh M Xu A Gamboa A Lemos T . Single-cell spatial analysis of tumor immune architecture in diffuse large B-cell lymphoma. Blood Adv. (2022) 6:4675–90. doi: 10.1182/bloodadvances.2022007493 35675517 PMC9631676

[52] Reiss DJ Nakayama Y Weng AP Stokes ME Sehn L Steidl C . High-plex imaging and cellular neighborhood spatial analysis reveals multiple immune escape and suppression patterns in diffuse large B-cell lymphoma. Leukemia. (2024) 38:1164–8. doi: 10.1038/s41375-024-02239-1 38575670 PMC11073958

[53] Autio M Leivonen S-K Meriranta L Karjalainen-Lindsberg M-L Pellinen T Leppä S . Spatial analysis of the tumor microenvironment in diffuse large B-cell lymphoma reveals clinically relevant cell interactions and recurrent cellular neighborhoods. Cancer Immunol Res. (2025) 13:1674–1686. doi: 10.1158/2326-6066.cir-24-1163 40772779 PMC12485370

[54] Abramson JS Solomon SR Arnason J Johnston PB Glass B Bachanova V . Lisocabtagene maraleucel as second-line therapy for large B-cell lymphoma: primary analysis of the phase 3 TRANSFORM study. Blood. (2023) 141:1675–84. doi: 10.1182/blood.2022018730 36542826 PMC10646768

[55] Neelapu SS Jacobson CA Ghobadi A Miklos DB Lekakis LJ Oluwole OO . Five-year follow-up of ZUMA-1 supports the curative potential of axicabtagene ciloleucel in refractory large B-cell lymphoma. Blood. (2023) 141:2307–15. doi: 10.1182/blood.2022018893 36821768 PMC10646788

[56] Schuster SJ Tam CS Borchmann P Worel N McGuirk JP Holte H . Long-term clinical outcomes of tisagenlecleucel in patients with relapsed or refractory aggressive B-cell lymphomas (JULIET): a multicentre, open-label, single-arm, phase 2 study. Lancet Oncol. (2021) 22:1403–15. doi: 10.1016/s1470-2045(21)00375-2 34516954

[57] Abramson JS Palomba ML Gordon LI Lunning M Wang M Arnason J . Two-year follow-up of lisocabtagene maraleucel in relapsed or refractory large B-cell lymphoma in TRANSCEND NHL 001. Blood. (2024) 143:404–16. doi: 10.1182/blood.2023020854 37890149

[58] Westin JR Oluwole OO Kersten MJ Miklos DB Perales M-A Ghobadi A . Survival with axicabtagene ciloleucel in large B-cell lymphoma. N Engl J Med. (2023) 389:148–57. doi: 10.1056/nejmoa2301665 37272527

[59] Bishop MR Dickinson M Purtill D Barba P Santoro A Hamad N . Second-line tisagenlecleucel or standard care in aggressive B-cell lymphoma. N Engl J Med. (2021) 386:629–39. doi: 10.1056/nejmoa2116596 34904798

[60] Administration UFaD . Cellular and Gene Therapy Products: YESCARTA (Axicabtagene Ciloleucel) 2024 (2025). Available online at: https://www.fda.gov/vaccines-blood-biologics/cellular-gene-therapy-products/yescarta (Accessed June 15, 2025).

[61] Dreger P Holtick U Subklewe M von Tresckow B Ayuk F Wagner E . Impact of age on outcome of CAR-T cell therapies for large B-cell lymphoma: the GLA/DRST experience. Bone Marrow Transplant. (2023) 58:229–32. doi: 10.1038/s41409-022-01867-4 36418916 PMC9902271

[62] Ram R Grisariu S Shargian-Alon L Amit O Bar-On Y Stepensky P . Toxicity and efficacy of chimeric antigen receptor T-cell therapy in patients with diffuse large B-cell lymphoma above the age of 70 years compared to younger patients – a matched control multicenter cohort study. Haematologica. (2022) 107:1111–8. doi: 10.3324/haematol.2021.278288 34233446 PMC9052918

[63] Jacobson CA Locke FL Ma L Asubonteng J Hu Z-H Siddiqi T . Real-world evidence of axicabtagene ciloleucel for the treatment of large B cell lymphoma in the United States. Transplant Cell Ther. (2022) 28:581.e1–.e8. doi: 10.1016/j.jtct.2022.05.026 35609867 PMC9427701

[64] Thiruvengadam SK Merryman R Wang Y Gaulin C Bezerra E Voorhees T . Outcomes of CD19 CAR T in transformed indolent lymphoma compared to de novo aggressive large B-cell lymphoma. Am J Hematol. (2025) 100:236–48. doi: 10.1002/ajh.27548 39715004 PMC11705210

[65] Thiruvengadam S Ahn KW Patel DJ Lian Q Shadman M Turtle CJ . CD19-directed CAR-T therapy for transformed follicular lymphoma: a CIBMTR analysis. Transplant Cell Ther. (2025) 31:S384–5. doi: 10.1016/j.jtct.2025.01.593 40762207 PMC12582634

[66] Nadiminti KV Ahn KW Patel J Lian Q Bezerra E Chen A . Chimeric antigen receptor T-cell therapy for Richter transformation: a CIBMTR analysis. Transplant Cell Ther. (2025) 31:1000.e1–1000.e11. doi: 10.1016/j.jtct.2025.07.021 40754223 PMC12718520

[67] Gauthier J Ahn KW Patel J Lian Q Badawy S Cairo MS . CD19 CAR T-cell therapy for primary mediastinal large B-cell lymphoma: a CIBMTR analysis. Am J Hematol. (2025) 100:1792–802. doi: 10.1002/ajh.70033 PMC12608806 40785644

[68] Pophali PA Fein JA Ahn KW Allbee-Johnson M Ahmed N Awan FT . CD19-directed CART therapy for T-cell/histiocyte–rich large B-cell lymphoma. Blood Adv. (2024) 8:5290–6. doi: 10.1182/bloodadvances.2024013863 38985302 PMC11497379

[69] Hossain NM Ahn KW Patel DJ Lian Q Shadman M Hamadani M . CAR T outcomes in patients with high-grade B-cell lymphoma not otherwise specified (HGBL-NOS): a CIBMTR analysis. Transplant Cell Ther. (2025) 31:S385–6. doi: 10.1016/j.jtct.2025.01.594 38826717

[70] Stephan P Di Blasi R Roulin L Galtier J Calvani J Meignin V . TRANSCAR: real-world outcomes of CD19 CAR T-cell therapy in relapsed/refractory transformed indolent lymphomas. Blood Adv. (2025) 9:4693–704. doi: 10.1182/bloodadvances.2025015834 40311067 PMC12466217

[71] Bensaber H Ferrant E Duléry R Beauvais D Feugier P Gastinne T . Anti-CD19 CAR T-cell therapy for patients with Richter transformation: a LYSA study from the DESCAR-T registry. Br J Haematol. (2025) 207:1143–7. doi: 10.1182/blood-2022-158807 40954116

[72] Galtier J Mesguich C Sesques P Dupont V Bachy E Di Blasi R . Outcomes of patients with relapsed or refractory primary mediastinal B-cell lymphoma treated with anti-CD19 CAR-T cells: CARTHYM, a study from the French national DESCAR-T registry. HemaSphere. (2025) 9:e70091. doi: 10.1002/hem3.70091 39968186 PMC11833168

[73] Phina-Ziebin X Bachy E Gros F-X Di Blasi R Herbaux C Bay JO . Outcome of high-grade B-cell lymphoma compared with other large B-cell lymphoma after CAR-T rescue: a DESCAR-T LYSA study. Blood Adv. (2025) 9:2500–10. doi: 10.1182/bloodadvances.2024014732 39874518 PMC12148394

[74] Hirayama AV Gauthier J Hay KA Voutsinas JM Wu Q Gooley T . The response to lymphodepletion impacts PFS in patients with aggressive non-Hodgkin lymphoma treated with CD19 CAR T cells. Blood. (2019) 133:1876–87. doi: 10.1182/blood-2018-11-887067 30782611 PMC6484391

[75] Raj SS Fei T Fried S Ip A Fein JA Leslie LA . An inflammatory biomarker signature of response to CAR-T cell therapy in non-Hodgkin lymphoma. Nat Med. (2025) 31:1183–94. doi: 10.1038/s41591-025-03532-x 40169864 PMC12003198

[76] Ghilardi G Paruzzo L Svoboda J Chong EA Shestov AA Chen L . Bendamustine lymphodepletion before axicabtagene ciloleucel is safe and associates with reduced inflammatory cytokines. Blood Adv. (2024) 8:653–66. doi: 10.1182/bloodadvances.2023011492 38113468 PMC10839610

[77] Bharadwaj S Lau E Hamilton MP Goyal A Srinagesh H Jensen A . Bendamustine is a safe and effective lymphodepletion agent for axicabtagene ciloleucel in patients with refractory or relapsed large B-cell lymphoma. J Immunother Cancer. (2024) 12:e008975. doi: 10.1136/jitc-2024-008975 38955420 PMC11218002

[78] Deschênes-Simard X Bromberg M Devlin SM Gonen M Beyar-Katz O Ip A . Comparative real-world outcomes of CD19-directed CAR T-cell therapies in large B-cell lymphoma. Blood Adv. (2025) 9:5571–84. doi: 10.1182/bloodadvances.2025016778 40815804 PMC12615284

[79] Jacobson CA Munoz J Sun F Kanters S Limbrick-Oldfield EH Spooner C . Real-world outcomes with chimeric antigen receptor T cell therapies in large B cell lymphoma: a systematic review and meta-analysis. Transplant Cell Ther. (2024) 30:77.e1–.e15. doi: 10.1016/j.jtct.2023.10.017 37890589

[80] Sommermeyer D Hudecek M Kosasih PL Gogishvili T Maloney DG Turtle CJ . Chimeric antigen receptor-modified T cells derived from defined CD8+ and CD4+ subsets confer superior antitumor reactivity *in vivo*. Leukemia. (2016) 30:492–500. doi: 10.1038/leu.2015.247 26369987 PMC4746098

[81] Monfrini C Stella F Aragona V Magni M Ljevar S Vella C . Phenotypic composition of commercial anti-CD19 CAR T cells affects *in vivo* expansion and disease response in patients with large B-cell lymphoma. Clin Cancer Res. (2022) 28:3378–86. doi: 10.1158/1078-0432.ccr-22-0164 35583610 PMC9662896

[82] Sehgal A Hoda D Riedell PA Ghosh N Hamadani M Hildebrandt GC . Lisocabtagene maraleucel as second-line therapy in adults with relapsed or refractory large B-cell lymphoma who were not intended for haematopoietic stem cell transplantation (PILOT): an open-label, phase 2 study. Lancet Oncol. (2022) 23:1066–77. doi: 10.1016/s1470-2045(22)00339-4 35839786

[83] Kuhnl A Roddie C Kirkwood AA Menne T Cuadrado M Marzolini MAV . Early FDG-PET response predicts CAR-T failure in large B-cell lymphoma. Blood Adv. (2022) 6:321–6. doi: 10.1182/bloodadvances.2021005807 34700342 PMC8753214

[84] Frank MJ Hossain NM Bukhari A Dean E Spiegel JY Claire GK . Monitoring of circulating tumor DNA improves early relapse detection after axicabtagene ciloleucel infusion in large B-cell lymphoma: results of a prospective multi-institutional trial. J Clin Oncol. (2021) 39:3034–43. doi: 10.1200/jco.21.00377 34133196 PMC10166351

[85] Bourlon C Roddie C Menne T Norman J O’Reilly M Gibb A . Outcomes after chimeric antigen receptor T-cell therapy across large B-cell lymphoma subtypes. Haematologica. (2024) 109:2716–20. doi: 10.3324/haematol.2024.285010 38572567 PMC11290504

[86] Alizadeh AA Eisen MB Davis RE Ma C Lossos IS Rosenwald A . Distinct types of diffuse large B-cell lymphoma identified by gene expression profiling. Nature. (2000) 403:503–11. doi: 10.1038/35000501 10676951

[87] Rosenwald A Wright G Chan WC Connors JM Campo E Fisher RI . The use of molecular profiling to predict survival after chemotherapy for diffuse large B-cell lymphoma. N Engl J Med. (2002) 346:1937–47. doi: 10.1017/cbo9780511663369.013 12075054

[88] Shipp MA Ross KN Tamayo P Weng AP Kutok JL Aguiar RCT . Diffuse large B-cell lymphoma outcome prediction by gene-expression profiling and supervised machine learning. Nat Med. (2002) 8:68–74. doi: 10.1038/nm0102-68 11786909

[89] Wright G Tan B Rosenwald A Hurt EH Wiestner A Staudt LM . A gene expression-based method to diagnose clinically distinct subgroups of diffuse large B cell lymphoma. Proc Natl Acad Sci. (2003) 100:9991–6. doi: 10.1073/pnas.1732008100 12900505 PMC187912

[90] Scott DW Wright GW Williams PM Lih C-J Walsh W Jaffe ES . Determining cell-of-origin subtypes of diffuse large B-cell lymphoma using gene expression in formalin-fixed paraffin-embedded tissue. Blood. (2014) 123:1214–21. doi: 10.1182/blood-2013-11-536433 24398326 PMC3931191

[91] Mottok A Wright G Rosenwald A Ott G Ramsower C Campo E . Molecular classification of primary mediastinal large B-cell lymphoma using routinely available tissue specimens. Blood. (2018) 132:2401–5. doi: 10.1182/blood-2018-05-851154 30257882 PMC6265647

[92] Ennishi D Jiang A Boyle M Collinge B Grande BM Ben-Neriah S . Double-hit gene expression signature defines a distinct subgroup of germinal center B-cell-like diffuse large B-cell lymphoma. J Clin Oncol. (2019) 37:190–201. doi: 10.1200/jco.18.01583 30523716 PMC6804880

[93] Hans CP Weisenburger DD Greiner TC Gascoyne RD Delabie J Ott G . Confirmation of the molecular classification of diffuse large B-cell lymphoma by immunohistochemistry using a tissue microarray. Blood. (2004) 103:275–82. doi: 10.1182/blood-2003-05-1545 14504078

[94] Sha C Barrans S Cucco F Bentley MA Care MA Cummin T . Molecular high-grade B-cell lymphoma: defining a poor-risk group that requires different approaches to therapy. J Clin Oncol. (2019) 37:202–12. doi: 10.1200/jco.18.01314 30523719 PMC6338391

[95] Olson NE Ragan SP Reiss DJ Thorpe J Kim Y Abramson JS . Exploration of tumor biopsy gene signatures to understand the role of the tumor microenvironment in outcomes to lisocabtagene maraleucel. Mol Cancer Ther. (2023) 22:406–18. doi: 10.1158/1535-7163.mct-21-0506 36595660 PMC9978882

[96] Sworder BJ Kurtz DM Alig SK Frank MJ Shukla N Garofalo A . Determinants of resistance to engineered T cell therapies targeting CD19 in large B cell lymphomas. Cancer Cell. (2023) 41:210–225.e5. doi: 10.1016/j.ccell.2022.12.005 36584673 PMC10010070

[97] Locke FL Filosto S Chou J Vardhanabhuti S Perbost R Dreger P . Impact of tumor microenvironment on efficacy of anti-CD19 CAR T cell therapy or chemotherapy and transplant in large B cell lymphoma. Nat Med. (2024) 30:507–18. doi: 10.1038/s41591-023-02754-1 38233586 PMC10878966

[98] Jain MD Ziccheddu B Coughlin CA Faramand R Griswold AJ Reid KM . Whole-genome sequencing reveals complex genomic features underlying anti-CD19 CAR T-cell treatment failures in lymphoma. Blood. (2022) 140:491–503. doi: 10.1182/blood.2021015008 35476848 PMC9353150

[99] Plaks V Rossi JM Chou J Wang L Poddar S Han G . CD19 target evasion as a mechanism of relapse in large B-cell lymphoma treated with axicabtagene ciloleucel. Blood. (2021) 138:1081–5. doi: 10.1182/blood.2021010930 34041526 PMC8462361

[100] Hernani R Ventura L Heras B Serrano A Rivada M Martínez-Ciarpaglini C . Clinical impact of CD19 expression assessed by quantitative PCR in lymphoma patients undergoing CAR-T therapy. Ejhaem. (2025) 6:e270015. doi: 10.1002/jha2.70015 40110072 PMC11920814

[101] Jain MD Zhao H Wang X Atkins R Menges M Reid K . Tumor interferon signaling and suppressive myeloid cells are associated with CAR T-cell failure in large B-cell lymphoma. Blood. (2021) 137:2621–33. doi: 10.1182/blood.2020007445 33512407 PMC8120145

[102] Hirayama AV Wright JH Smythe KS Fiorenza S Shaw AN Gauthier J . PD-L1(+) macrophage and tumor cell abundance and proximity to T cells in the pretreatment large B-cell lymphoma microenvironment impact CD19 CAR-T cell immunotherapy efficacy. Hemasphere. (2024) 8:e142. doi: 10.1002/hem3.142 39113729 PMC11303978

[103] Majzner RG Frank MJ Mount C Tousley A Kurtz DM Sworder B . CD58 aberrations limit durable responses to CD19 CAR in large B cell lymphoma patients treated with axicabtagene ciloleucel but can be overcome through novel CAR engineering. Blood. (2020) 136:53–4. doi: 10.1182/blood-2020-139605

[104] Roider T Baertsch MA Fitzgerald D Vöhringer H Brinkmann BJ Czernilofsky F . Multimodal and spatially resolved profiling identifies distinct patterns of T cell infiltration in nodal B cell lymphoma entities. Nat Cell Biol. (2024) 26:478–89. doi: 10.1038/s41556-024-01358-2 38379051 PMC10940160

[105] Chen P-H Lipschitz M Weirather JL Jacobson C Armand P Wright K . Activation of CAR and non-CAR T cells within the tumor microenvironment following CAR T cell therapy. JCI Insight. (2020) 5:e134612. doi: 10.1172/jci.insight.134612 32484797 PMC7406247

[106] Jin J Lin L Meng J Jiang L Zhang M Fang Y . High-multiplex single-cell imaging analysis reveals tumor immune contexture associated with clinical outcomes after CAR T-cell therapy. Mol Ther. (2024) 32:1252–1265. doi: 10.1016/j.ymthe.2024.03.023 38504519 PMC11081919

[107] Yan Z-X Li L Wang W OuYang B-S Cheng S Wang L . Clinical efficacy and tumor microenvironment influence in a dose-escalation study of anti-CD19 chimeric antigen receptor T cells in refractory B-cell non-Hodgkin's lymphoma. Clin Cancer Res. (2019) 25:6995–7003. doi: 10.1158/1078-0432.ccr-19-0101 31444250

[108] Pagès F Mlecnik B Marliot F Bindea G Ou F-S Bifulco C . International validation of the consensus Immunoscore for the classification of colon cancer: a prognostic and accuracy study. Lancet. (2018) 391:2128–39. doi: 10.1016/S0140-6736(18)30789-X 29754777

[109] Scholler N Perbost R Locke FL Jain MD Turcan S Danan C . Tumor immune contexture is a determinant of anti-CD19 CAR T cell efficacy in large B cell lymphoma. Nat Med. (2022) 28:1872–82. doi: 10.1038/s41591-022-01916-x 36038629 PMC9499856

[110] Yan Z Li L Fu D Wu W Qiao N Huang Y . Immunosuppressive tumor microenvironment contributes to tumor progression in diffuse large B-cell lymphoma upon anti-CD19 chimeric antigen receptor T therapy. Front Med. (2023) 17:699–713. doi: 10.1007/s11684-022-0972-8 37060525

[111] Cervantes EV Boucher JC Lee SB Spitler K Reid K Davila ML . MDSC suppression of CAR T cells can be reduced by targeted signaling disruption. Blood. (2019) 134:4438. doi: 10.1182/blood-2019-122752

[112] Boulch M Cazaux M Loe-Mie Y Thibaut R Corre B Lemaître F . A cross-talk between CAR T cell subsets and the tumor microenvironment is essential for sustained cytotoxic activity. Sci Immunol. (2021) 6:eabd4344. doi: 10.1126/sciimmunol.abd4344 33771887

[113] Cazaux M Grandjean CL Lemaître F Garcia Z Beck RJ Milo I . Single-cell imaging of CAR T cell activity *in vivo* reveals extensive functional and anatomical heterogeneity. J Exp Med. (2019) 216:1038–49. doi: 10.1084/jem.20182375 30936262 PMC6504219

[114] Chapuy B Stewart C Dunford AJ Kim J Kamburov A Redd RA . Molecular subtypes of diffuse large B cell lymphoma are associated with distinct pathogenic mechanisms and outcomes. Nat Med. (2018) 24:679–90. doi: 10.1038/s41591-018-0016-8 29713087 PMC6613387

[115] Wright GW Huang DW Phelan JD Coulibaly ZA Roulland S Young RM . A probabilistic classification tool for genetic subtypes of diffuse large B cell lymphoma with therapeutic implications. Cancer Cell. (2020) 37:551–568.e14. doi: 10.1016/j.ccell.2020.03.015 32289277 PMC8459709

[116] Schmitz R Wright GW Huang DW Johnson CA Phelan JD Wang JQ . Genetics and pathogenesis of diffuse large B-cell lymphoma. N Engl J Med. (2018) 378:1396–407. doi: 10.1056/nejmoa1801445 29641966 PMC6010183

[117] Shi H Zheng P Liu R Xu T Yang F Feng S . Genetic landscapes and curative effect of CAR T-cell immunotherapy in patients with relapsed or refractory DLBCL. Blood Adv. (2023) 7:1070–5. doi: 10.1182/bloodadvances.2021006845 35901280 PMC10034568

[118] Hill BT Roth CJ Kositsky R Dave T Love C McKinney M . Impact of molecular features of diffuse large B-cell lymphoma on treatment outcomes with anti-CD19 chimeric antigen receptor (CAR) T-cell therapy. Blood. (2021) 138:165. doi: 10.1182/blood-2021-145764

[119] Shouval R Tomas AA Fein JA Flynn JR Markovits E Mayer S . Impact of TP53 genomic alterations in large B-cell lymphoma treated with CD19-chimeric antigen receptor T-cell therapy. J Clin Oncol. (2022) 40:369–81. doi: 10.1200/jco.21.02143 34860572 PMC8797602

[120] Rossi JM Galon J Turcan S Danan C Locke FL Neelapu SS . Clinical response in ZUMA-1, the pivotal study of axicabtagene ciloleucel (Axi-Cel) in patients with refractory large B cell lymphoma, may be influenced by characteristics of the pretreatment tumor microenvironment (TME). Clin Lymphoma Myeloma Leukemia. (2018) 18:S281. doi: 10.26226/morressier.5b3b802db1b87b000eceda50

[121] Benci JL Xu B Qiu Y Wu TJ Dada H Twyman-Saint Victor C . Tumor interferon signaling regulates a multigenic resistance program to immune checkpoint blockade. Cell. (2016) 167:1540–1554.e12. doi: 10.1016/j.cell.2016.11.022 27912061 PMC5385895

[122] Good Z Spiegel JY Sahaf B Malipatlolla MB Ehlinger ZJ Kurra S . Post-infusion CAR TReg cells identify patients resistant to CD19-CAR therapy. Nat Med. (2022) 28:1860–71. doi: 10.1038/s41591-022-01960-7 36097223 PMC10917089

[123] Gurevich-Shapiro A Zwicky P Winter E Zada M Shapira N Barboy O . Distinct cellular and molecular patterns in pretreatment peripheral blood are associated with CAR T-cell outcomes in diffuse large B-cell lymphoma. Cancer Res. (2025) 85:5066–83. doi: 10.1158/0008-5472.can-25-3596 41071708

[124] Huang S Wang X Wang Y Wang Y Fang C Wang Y . Deciphering and advancing CAR T-cell therapy with single-cell sequencing technologies. Mol Cancer. (2023) 22:80. doi: 10.1186/s12943-023-01783-1 37149643 PMC10163813

[125] Rezvan A Romain G Fathi M Heeke D Martinez-Paniagua M An X . Identification of a clinically efficacious CAR T cell subset in diffuse large B cell lymphoma by dynamic multidimensional single-cell profiling. Nat Cancer. (2024) 5:1010–23. doi: 10.1038/s43018-024-00768-3 38750245 PMC12345446

[126] Fiorenza S Zheng Y Purushe J Bock TJ Sarthy J Janssens DH . Histone marks identify novel transcription factors that parse CAR-T subset-of-origin, clinical potential and expansion. Nat Commun. (2024) 15:8309. doi: 10.1038/s41467-024-52503-2 39333103 PMC11436946

[127] Williams CG Lee HJ Asatsuma T Vento-Tormo R Haque A . An introduction to spatial transcriptomics for biomedical research. Genome Med. (2022) 14:68. doi: 10.1186/s13073-022-01075-1 35761361 PMC9238181

[128] Dai Y Kizhakeyil A Chihara D Li X Liu Y Sainz Zuniga TP . Multi-modal spatial characterization of tumor immune microenvironments identifies targetable inflammatory niches in diffuse large B cell lymphoma. Nat Genet. (2025) 57:2715–27. doi: 10.1038/s41588-025-02353-5 41120574 PMC12597830

[129] Dai L Lou N Huang L Li L Tang L Shi Y . Spatial transcriptomics reveals prognostically LYZ+ fibroblasts and colocalization with FN1+ macrophages in diffuse large B-cell lymphoma. Cancer Immunol Immunother. (2025) 74:123. doi: 10.1007/s00262-025-03968-7 39998673 PMC11861843

[130] Dai L Fan G Xie T Li L Tang L Chen H . Single-cell and spatial transcriptomics reveal a high glycolysis B cell and tumor-associated macrophages cluster correlated with poor prognosis and exhausted immune microenvironment in diffuse large B-cell lymphoma. biomark Res. (2024) 12:58. doi: 10.1186/s40364-024-00605-w 38840205 PMC11155084

[131] Lou N Dai L Gao R Yang J Gui L Yang S . Single-cell sequencing and spatial transcriptomics reveal FAS+ T cell and autophagy-related signatures predicting chemoimmunotherapy response in diffuse large B-cell lymphoma patients. Sci China Life Sci. (2025) 68:2316–2331. doi: 10.1007/s11427-024-2849-2 40374987

[132] Liu M Bertolazzi G Sridhar S Lee RX Jaynes P Mulder K . Spatially-resolved transcriptomics reveal macrophage heterogeneity and prognostic significance in diffuse large B-cell lymphoma. Nat Commun. (2024) 15:2113. doi: 10.1038/s41467-024-46220-z 38459052 PMC10923916

[133] Sangaletti S Iannelli F Zanardi F Cancila V Portararo P Botti L . Intra-tumour heterogeneity of diffuse large B-cell lymphoma involves the induction of diversified stroma-tumour interfaces. Ebiomedicine. (2020) 61:103055. doi: 10.1016/j.ebiom.2020.103055 33096480 PMC7581880

[134] Xia Y Sun T Li G Li M Wang D Su X . Spatial single cell analysis of tumor microenvironment remodeling pattern in primary central nervous system lymphoma. Leukemia. (2023) 37:1499–510. doi: 10.1038/s41375-023-01908-x 37120690 PMC10317840

[135] Heming M Haessner S Wolbert J Lu IN Li X Brokinkel B . Intratumor heterogeneity and T cell exhaustion in primary CNS lymphoma. Genome Med. (2022) 14:109. doi: 10.1186/s13073-022-01110-1 36153593 PMC9509601

[136] Abe Y Zenkoh J Kanai A Ikeda D Kaji D Sawa A . Distinct follicular T cell subsets regulate lymphoma progression and outcomes. Cancer Cell. (2025) 43:1850–1865.e11. doi: 10.1016/j.ccell.2025.06.013 40614737

[137] Liu H Lei W Zhang C Yang C Wei J Guo Q . CD19-specific CAR T cells that express a PD-1/CD28 chimeric switch-receptor are effective in patients with PD-L1⇓positive B-cell lymphoma. Clin Cancer Res. (2021) 27:473–84. doi: 10.1158/1078-0432.ccr-20-1457 33028589

[138] Hu Y Zu C Zhang M Wei G Li W Fu S . Safety and efficacy of CRISPR-based non-viral PD1 locus specifically integrated anti-CD19 CAR-T cells in patients with relapsed or refractory Non-Hodgkin's lymphoma: a first-in-human phase I study. eClinicalMedicine. (2023) 60:102010. doi: 10.1016/j.eclinm.2023.102010 37251628 PMC10209187

[139] Kim W Kim SJ Yoon DH Eom H Yang D Yoon SE . Phase 2 study of ANBAL-CEL, novel anti-CD19 CAR-T therapy with dual silencing of PD-1 and TIGIT in relapsed or refractory large B cell lymphoma - interim analysis result. Hematological Oncol. (2023) 41:81–2. doi: 10.1097/01.hs9.0000843748.64635.ac 33079766

[140] Liu X Zhang Y Li K Liu Y Xu J Ma J . A novel dominant-negative PD-1 armored anti-CD19 CAR T cell is safe and effective against refractory/relapsed B cell lymphoma. Transl Oncol. (2021) 14:101085. doi: 10.1016/j.tranon.2021.101085 33813229 PMC8050776

[141] Hu B Nastoupil LJ Holmes H Hamdan A Kanate A Farooq U . A CRISPR-edited allogeneic anti-CD19 CAR-T cell therapy with a PD-1 knockout (CB-010) in patients with relapsed/refractory B cell non-Hodgkin lymphoma (r/r B-NHL): Updated phase 1 results from the ANTLER trial. J Clin Oncol. (2024) 42:7025. doi: 10.1200/jco.2024.42.16_suppl.7025 42148471

[142] Wang Z Xu H Mei Y Xiao M Cao Y Huang L . Combination of chidamide and PD-1 blockade in refractory/relapsed aggressive large B-cell lymphomas with high risk of failing CAR-T therapy. Int Immunopharmacol. (2024) 133:112014. doi: 10.1016/j.intimp.2024.112014 38615378

[143] Hirayama AV Kimble EL Wright JH Fiorenza S Gauthier J Voutsinas JM . Timing of anti–PD-L1 antibody initiation affects efficacy/toxicity of CD19 CAR T-cell therapy for large B-cell lymphoma. Blood Adv. (2024) 8:453–67. doi: 10.1182/bloodadvances.2023011287 37903325 PMC10837185

[144] Jaeger U Worel N McGuirk JP Riedell PA Fleury I Du Y . Safety and efficacy of tisagenlecleucel plus pembrolizumab in patients with r/r DLBCL: phase 1b PORTIA study results. Blood Adv. (2023) 7:2283–6. doi: 10.1182/bloodadvances.2022007779 36044388 PMC10225880

[145] Xue B Luo X Liu Y Ye S Zhou L Li S . CAR T-cell therapy combined with PD-1 inhibitors significantly improve the efficacy and prognosis of r/r DLBCL with TP53 alterations. Blood. (2023) 142:3515. doi: 10.1182/blood-2023-180500

[146] Mu J Deng H Lyu C Yuan J Li Q Wang J . Efficacy of programmed cell death 1 inhibitor maintenance therapy after combined treatment with programmed cell death 1 inhibitors and anti-CD19-chimeric antigen receptor T cells in patients with relapsed/refractory diffuse large B-cell lymphoma and high tumor burden. Hematol Oncol. (2023) 41:275–84. doi: 10.1002/hon.2981 35195933

[147] Jacobson CA Westin JR Miklos DB Herrera AF Lee J Seng J . Abstract CT055: Phase 1/2 primary analysis of ZUMA-6: Axicabtagene ciloleucel (Axi-Cel) in combination with atezolizumab (Atezo) for the treatment of patients (Pts) with refractory diffuse large B cell lymphoma (DLBCL). Cancer Res. (2020) 80:CT055. doi: 10.1158/1538-7445.am2020-ct055 36230740

[148] Cao Y Lu W Sun R Jin X Cheng L He X . Anti-CD19 chimeric antigen receptor T cells in combination with nivolumab are safe and effective against relapsed/refractory B-cell non-hodgkin lymphoma. Front Oncol. (2019) 9. doi: 10.3389/fonc.2019.00767 31482064 PMC6709654

[149] Siddiqi T Abramson JS Lee HJ Schuster S Hasskarl J Montheard S . Safety of lisocabtagene maraleucel given with durvalumab in patients with relapsed/refractory aggressive B‐cell non hodgkin lymphoma: first results from the PLATFORM study. Hematological Oncol. (2019) 37:171–2. doi: 10.1002/hon.128_2629

[150] Osborne W Marzolini M Tholouli E Ramakrishnan A Bachier CR McSweeney PA . Phase I Alexander study of AUTO3, the first CD19/22 dual targeting CAR T cell therapy, with pembrolizumab in patients with relapsed/refractory (r/r) DLBCL. J Clin Oncol. (2020) 38:8001. doi: 10.1200/jco.2020.38.15_suppl.8001 42148471

[151] Ahmed S DiPersio JF Essell JH Diefenbach CS Perales MA Castilla-Llorente C . NKTR-255 vs placebo to enhance complete responses and durability following CD19-directed CAR-T therapy in patients with relapsed/ refractory (R/R) large B-cell lymphoma (LBCL). Blood. (2024) 144:2068. doi: 10.1182/blood-2024-203576

[152] Svoboda J Landsburg DJ Gerson J Nasta SD Barta SK Chong EA . Enhanced CAR T-cell therapy for lymphoma after previous failure. N Engl J Med. (2025) 392:1824–35. doi: 10.1056/nejmoa2408771 40334157

[153] Lei W Zhao A Liu H Yang C Wei C Guo S . Safety and feasibility of anti-CD19 CAR T cells expressing inducible IL-7 and CCL19 in patients with relapsed or refractory large B-cell lymphoma. Cell Discov. (2024) 10:5. doi: 10.21203/rs.3.rs-2124394/v1 38191529 PMC10774422

[154] Palomba ML Caimi PF Mei M Hernandez-Ilizaliturri F Shouse G Winter AM . A phase 1 study to evaluate the safety and tolerability of a combination autologous CD19 CAR T cell therapy (SYNCAR-001) and orthogonal IL-2 (STK-009) in subjects with relapsed or refractory CD19 expressing hematologic Malignancies (NCT05665062). Blood. (2024) 144:3453. doi: 10.1182/blood-2024-202090

[155] Armengol M Santos JC Fernández-Serrano M Profitós-Pelejà N Ribeiro ML Roué G . Immune-checkpoint inhibitors in B-cell lymphoma. Cancers. (2021) 13:214. doi: 10.3390/cancers13020214 33430146 PMC7827333

[156] Chong EA Melenhorst JJ Svoboda J Dwivedy Nasta S Landsburg DJ Mato AR . Phase I/II study of pembrolizumab for progressive diffuse large B cell lymphoma after anti-CD19 directed chimeric antigen receptor modified T cell therapy. Blood. (2017) 130:4121.

[157] Chong EA Alanio C Svoboda J Nasta SD Landsburg DJ Lacey SF . Pembrolizumab for B-cell lymphomas relapsing after or refractory to CD19-directed CAR T-cell therapy. Blood. (2022) 139:1026–38. doi: 10.1182/blood.2021012634 34496014 PMC9211527

[158] Zheng B Hu Y Zhang J Zhang M Li W Wu W . Long term follow-up results of Brl-201 phase I study, a crispr-based non-viral PD-1 locus specific integrated anti-CD19 CAR-T cells in treating relapsed or refractory non-Hodgkin's lymphoma. Blood. (2023) 142:2108. doi: 10.1182/blood-2023-185573

[159] Zurko J Chaney K Astle JM Johnson BD Hari P Shah NN . PD-1 blockade after bispecific LV20.19 CAR T modulates CAR T-cell immunophenotype without meaningful clinical response. Haematologica. (2021) 106:2788–90. doi: 10.3324/haematol.2021.278461 33853295 PMC8485669

[160] Chong EA Svoboda J Dwivedy Nasta S Landsburg DJ Winchell N Napier E . Sequential anti-CD19 directed chimeric antigen receptor modified T-cell therapy (CART19) and PD-1 blockade with pembrolizumab in patients with relapsed or refractory B-cell non-Hodgkin lymphomas. Blood. (2018) 132:4198. doi: 10.1182/blood-2018-99-119502

[161] Zhang H Li F Cao J Wang X Cheng H Qi K . A chimeric antigen receptor with antigen-independent OX40 signaling mediates potent antitumor activity. Sci Transl Med. (2021) 13:eaba7308. doi: 10.1126/scitranslmed.aba7308 33504651

[162] Kastenschmidt JM Schroers-Martin JG Sworder BJ Sureshchandra S Khodadoust MS Liu CL . A human lymphoma organoid model for evaluating and targeting the follicular lymphoma tumor immune microenvironment. Cell Stem Cell. (2024) 31:410–420.e4. doi: 10.1016/j.stem.2024.01.012 38402619 PMC10960522

[163] Agarwal S Aznar MA Rech AJ Good CR Kuramitsu S Da T . Deletion of the inhibitory co-receptor CTLA-4 enhances and invigorates chimeric antigen receptor T cells. Immunity. (2023) 56:2388–2407.e9. doi: 10.1016/j.immuni.2023.09.001 37776850 PMC10591801

[164] Zhang Y Zhang X Cheng C Mu W Liu X Li N . CRISPR-Cas9 mediated LAG-3 disruption in CAR-T cells. Front Med. (2017) 11:554–62. doi: 10.1007/s11684-017-0543-6 28625015

[165] Zhao S Wang C Lu P Lou Y Liu H Wang T . Switch receptor T3/28 improves long-term persistence and antitumor efficacy of CAR-T cells. J Immunother Cancer. (2021) 9:e003176. doi: 10.1136/jitc-2021-003176 34853180 PMC8638458

[166] Blaeschke F Ortner E Stenger D Mahdawi J Apfelbeck A Habjan N . Design and evaluation of TIM-3-CD28 checkpoint fusion proteins to improve anti-CD19 CAR T-cell function. Front Immunol. (2022) 13. doi: 10.3389/fimmu.2022.845499 35464394 PMC9018974

[167] Falgàs A Lázaro-Gorines R Zanetti SR Rubio-Pérez L Martínez-Moreno A Vinyoles M . A TIM-3–Fc decoy secreted by engineered T cells improves CD19 CAR T-cell therapy in B-cell acute lymphoblastic leukemia. Blood. (2025) 145:2599–613. doi: 10.1182/blood.2024025440 40090006

[168] Lee Y-H Lee HJ Kim HC Lee Y Nam SK Hupperetz C . PD-1 and TIGIT downregulation distinctly affect the effector and early memory phenotypes of CD19-targeting CAR T cells. Mol Ther. (2022) 30:579–92. doi: 10.1016/j.ymthe.2021.10.004 34628052 PMC8821960

[169] Khaniya A Khuisangeam N Tawinwung S Suppipat K Hirankarn N . A bidirectional EF1 promoter system for armoring CD19 CAR-T cells with secreted anti-PD1 antibodies. Int J Mol Sci. (2025) 26:11566. doi: 10.3390/ijms262311566 41373716 PMC12692150

[170] Hoyos V Savoldo B Quintarelli C Mahendravada A Zhang M Vera J . Engineering CD19-specific T lymphocytes with interleukin-15 and a suicide gene to enhance their anti-lymphoma/leukemia effects and safety. Leukemia. (2010) 24:1160–70. doi: 10.1038/leu.2010.75 20428207 PMC2888148

[171] Hirayama AV Chou CK Miyazaki T Steinmetz RN Di HA Fraessle SP . A novel polymer-conjugated human IL-15 improves efficacy of CD19-targeted CAR T-cell immunotherapy. Blood Adv. (2023) 7:2479–93. doi: 10.1182/bloodadvances.2022008697 36332004 PMC10242497

[172] Sánchez-Moreno I Lasarte-Cia A Martín-Otal C Casares N Navarro F Gorraiz M . Tethered IL15-IL15Rα augments antitumor activity of CD19 CAR-T cells but displays long-term toxicity in an immunocompetent lymphoma mouse model. J Immunother Cancer. (2024) 12:e008572. doi: 10.1136/jitc-2023-008572 38955421 PMC11218034

[173] Štach M Ptáčková P Mucha M Musil J Klener P Otáhal P . Inducible secretion of IL-21 augments anti-tumor activity of piggyBac-manufactured chimeric antigen receptor T cells. Cytotherapy. (2020) 22:744–54. doi: 10.1016/j.jcyt.2020.08.005 32950390

[174] Markley JC Sadelain M . IL-7 and IL-21 are superior to IL-2 and IL-15 in promoting human T cell–mediated rejection of systemic lymphoma in immunodeficient mice. Blood. (2010) 115:3508–19. doi: 10.1182/blood-2009-09-241398 20190192 PMC2867264

[175] Pegram HJ Lee JC Hayman EG Imperato GH Tedder TF Sadelain M . Tumor-targeted T cells modified to secrete IL-12 eradicate systemic tumors without need for prior conditioning. Blood. (2012) 119:4133–41. doi: 10.1182/blood-2011-12-400044 22354001 PMC3359735

[176] Li X Daniyan AF Lopez AV Purdon TJ Brentjens RJ . Cytokine IL-36γ improves CAR T-cell functionality and induces endogenous antitumor response. Leukemia. (2021) 35:506–21. doi: 10.1038/s41375-020-0874-1 32447345 PMC7680719

[177] Noh K-E Lee J-H Choi S-Y Jung N-C Nam J-H Oh J-S . TGF-β/IL-7 chimeric switch receptor-expressing CAR-T cells inhibit recurrence of CD19-positive B cell lymphoma. Int J Mol Sci. (2021) 22:8706. doi: 10.3390/ijms22168706 34445415 PMC8395772

[178] Zhou W Miao J Cheng Z Wang Z Wang J Guo H . Hypoxia-regulated secretion of IL-12 enhances antitumor activity and safety of CD19 CAR-T cells in the treatment of DLBCL. Mol Ther Oncolytics. (2023) 30:216–26. doi: 10.1016/j.omto.2023.08.009 37663131 PMC10471514

[179] Ninomiya S Narala N Huye L Yagyu S Savoldo B Dotti G . Tumor indoleamine 2,3-dioxygenase (IDO) inhibits CD19-CAR T cells and is downregulated by lymphodepleting drugs. Blood. (2015) 125:3905–16. doi: 10.1182/blood-2015-01-621474 25940712 PMC4473118

[180] Fultang L Booth S Yogev O Martins da Costa B Tubb V Panetti S . Metabolic engineering against the arginine microenvironment enhances CAR-T cell proliferation and therapeutic activity. Blood. (2020) 136:1155–60. doi: 10.1182/blood.2019004500 32573723 PMC7565134

[181] Vigano S Alatzoglou D Irving M Ménétrier-Caux C Caux C Romero P . Targeting adenosine in cancer immunotherapy to enhance T-cell function. Front Immunol. (2019) 10. doi: 10.3389/fimmu.2019.00925 31244820 PMC6562565

[182] Sakemura R Hefazi M Siegler EL Cox MJ Larson DP Hansen MJ . Targeting cancer-associated fibroblasts in the bone marrow prevents resistance to CART-cell therapy in multiple myeloma. Blood. (2022) 139:3708–21. doi: 10.1182/blood.2021012811 35090171 PMC11290597

[183] Yoon SE Kang W Cho J Cho HJ Chalita M Oh H-S . Microbiome and metabolite biomarkers of CAR T-cell therapy outcomes in relapsed/refractory diffuse large B-cell lymphoma. Blood Adv. (2026) 10:1634–45. doi: 10.1182/bloodadvances.2025016858 41364878 PMC12955626

